# Supplementary material for: Heuristic energy-based cyclic peptide design
Source: PLoS Comput Biol. 2025 Apr 30;21(4):e1012290. doi: 10.1371/journal.pcbi.1012290 (PMC12043242; doi:10.1371/journal.pcbi.1012290)
Supplement: S1 Text — (PDF) [file pcbi.1012290.s001.pdf]

1 Cyclic error derivation

Recall that a point with coordinate $\boldsymbol{r_{i+1}}$ in system $i+1$ has coordinate $\boldsymbol{r_i}$ in system i, following

$$\boldsymbol{r_i} = \boldsymbol{T_{\theta_{i+1}}} \boldsymbol{R_{\varphi_{i+1}}} \boldsymbol{r_{i+1}} + \boldsymbol{p_i}, \tag{1}$$

where

$$\boldsymbol{T_{\theta_{i+1}}} = \begin{bmatrix} cos(\pi - \theta_{i+1}) & -sin(\pi - \theta_{i+1}) & 0 \\ sin(\pi - \theta_{i+1}) & cos(\pi - \theta_{i+1}) & 0 \\ 0 & 0 & 1 \end{bmatrix},$$

$$\boldsymbol{R_{\varphi_{i+1}}} = \begin{bmatrix} 1 & 0 & 0 \\ 0 & cos(\varphi_{i+1}) & -sin(\varphi_{i+1}) \\ 0 & sin(\varphi_{i+1}) & cos(\varphi_{i+1}) \end{bmatrix}, \boldsymbol{p_i} = \begin{bmatrix} d_i \\ 0 \\ 0 \end{bmatrix}.$$

By choosing system 1 to be at atom C^α of residue n, we have bond lengths

$$\begin{aligned} d_1 = d_4 = d_7 = \cdots = d_{3n-2} = d_{3n+1} = d_{C^\alpha} \quad &(\text{bond length } C^\alpha\text{-}C'), \\ d_2 = d_5 = d_8 = \cdots = d_{3n-1} = d_{3n+2} = d_{C'} \quad &(\text{bond length } C'\text{-N}), \\ d_3 = d_6 = d_9 = \cdots = d_{3n} = d_{3n+3} = d_N \quad &(\text{bond length N-}C^\alpha), \end{aligned} \tag{2}$$

bond angles

$$\begin{aligned} \theta_1 = \theta_4 = \theta_7 = \cdots = \theta_{3n-2} = \theta_{3n+1} = \theta_{C^\alpha} \quad &(\text{bond angle N-}C^\alpha\text{-}C'), \\ \theta_2 = \theta_5 = \theta_8 = \cdots = \theta_{3n-1} = \theta_{3n+2} = \theta_{C'} \quad &(\text{bond angle } C^\alpha\text{-}C'\text{-N}), \\ \theta_3 = \theta_6 = \theta_9 = \cdots = \theta_{3n} = \theta_{3n+3} = \theta_N \quad &(\text{bond angle } C'\text{-N-}C^\alpha), \end{aligned} \tag{3}$$

and torsion angles

$$\begin{aligned} &\varphi_1 = \psi_n, \quad \varphi_4 = \psi_1, \quad \varphi_7 = \psi_2, \quad \cdots, \quad \varphi_{3n-2} = \psi_{n-1}, \quad \varphi_{3n+1} = \psi_n, \\ &\varphi_2 = \omega, \quad \varphi_5 = \omega, \quad \varphi_8 = \omega, \quad \cdots, \quad \varphi_{3n-1} = \omega, \quad \varphi_{3n+2} = \omega, \\ &\varphi_3 = \phi_1, \quad \varphi_6 = \phi_2, \quad \varphi_9 = \phi_3, \quad \cdots, \quad \varphi_{3n} = \phi_n, \quad \varphi_{3n+3} = \phi_1, \end{aligned} \tag{4}$$

where ϕ_j and ψ_j are torsion angles of residue j.

The origin of system $3n+1$ has coordinate $\boldsymbol{r_{3n+1}} = \boldsymbol{0}$ in system $3n+1$. Using Eq (1), Eq (2), Eq (3), and Eq (4), we have

$$\boldsymbol{r_{3n}} = \begin{bmatrix} d_N \\ 0 \\ 0 \end{bmatrix}, \quad \boldsymbol{r_{3n-1}} = \boldsymbol{T_{\theta_N}} \boldsymbol{R_{\phi_n}} \begin{bmatrix} d_N \\ 0 \\ 0 \end{bmatrix} + \begin{bmatrix} d_{C'} \\ 0 \\ 0 \end{bmatrix} = \boldsymbol{T_{\theta_N}} \begin{bmatrix} d_N \\ 0 \\ 0 \end{bmatrix} + \begin{bmatrix} d_{C'} \\ 0 \\ 0 \end{bmatrix},$$

$$\boldsymbol{r_{3n-2}} = \boldsymbol{T_{\theta_{C'}}} \boldsymbol{R_\omega} \boldsymbol{T_{\theta_N}} \begin{bmatrix} d_N \\ 0 \\ 0 \end{bmatrix} + \boldsymbol{T_{\theta_{C'}}} \begin{bmatrix} d_{C'} \\ 0 \\ 0 \end{bmatrix} + \begin{bmatrix} d_{C^\alpha} \\ 0 \\ 0 \end{bmatrix},$$

$$\boldsymbol{r_{3n-3}} = \boldsymbol{T_{\theta_{C^\alpha}}} \boldsymbol{R_{\psi_{n-1}}} \boldsymbol{T_{\theta_{C'}}} \boldsymbol{R_\omega} \boldsymbol{T_{\theta_N}} \begin{bmatrix} d_N \\ 0 \\ 0 \end{bmatrix} + \boldsymbol{T_{\theta_{C^\alpha}}} \boldsymbol{R_{\psi_{n-1}}} \boldsymbol{T_{\theta_{C'}}} \begin{bmatrix} d_{C'} \\ 0 \\ 0 \end{bmatrix} + \boldsymbol{T_{\theta_{C^\alpha}}} \begin{bmatrix} d_{C^\alpha} \\ 0 \\ 0 \end{bmatrix} + \begin{bmatrix} d_N \\ 0 \\ 0 \end{bmatrix},$$

$$\begin{aligned} \boldsymbol{r_{3n-4}} =& \boldsymbol{T_{\theta_N}} \boldsymbol{R_{\phi_{n-1}}} \boldsymbol{T_{\theta_{C^\alpha}}} \boldsymbol{R_{\psi_{n-1}}} \boldsymbol{T_{\theta_{C'}}} \boldsymbol{R_\omega} \boldsymbol{T_{\theta_N}} \begin{bmatrix} d_N \\ 0 \\ 0 \end{bmatrix} + \boldsymbol{T_{\theta_N}} \boldsymbol{R_{\phi_{n-1}}} \boldsymbol{T_{\theta_{C^\alpha}}} \boldsymbol{R_{\psi_{n-1}}} \boldsymbol{T_{\theta_{C'}}} \begin{bmatrix} d_{C'} \\ 0 \\ 0 \end{bmatrix} \\ &+ \boldsymbol{T_{\theta_N}} \boldsymbol{R_{\phi_{n-1}}} \boldsymbol{T_{\theta_{C^\alpha}}} \begin{bmatrix} d_{C^\alpha} \\ 0 \\ 0 \end{bmatrix} + \boldsymbol{T_{\theta_N}} \begin{bmatrix} d_N \\ 0 \\ 0 \end{bmatrix} + \begin{bmatrix} d_{C'} \\ 0 \\ 0 \end{bmatrix}, \end{aligned}$$

$$\begin{aligned} \boldsymbol{r_{3n-5}} =& \boldsymbol{T_{\theta_{C'}}} \boldsymbol{R_\omega} \boldsymbol{T_{\theta_N}} \boldsymbol{R_{\phi_{n-1}}} \boldsymbol{T_{\theta_{C^\alpha}}} \boldsymbol{R_{\psi_{n-1}}} \boldsymbol{T_{\theta_{C'}}} \boldsymbol{R_\omega} \boldsymbol{T_{\theta_N}} \begin{bmatrix} d_N \\ 0 \\ 0 \end{bmatrix} + \boldsymbol{T_{\theta_{C'}}} \boldsymbol{R_\omega} \boldsymbol{T_{\theta_N}} \boldsymbol{R_{\phi_{n-1}}} \boldsymbol{T_{\theta_{C^\alpha}}} \boldsymbol{R_{\psi_{n-1}}} \boldsymbol{T_{\theta_{C'}}} \begin{bmatrix} d_{C'} \\ 0 \\ 0 \end{bmatrix} \\ &+ \boldsymbol{T_{\theta_{C'}}} \boldsymbol{R_\omega} \boldsymbol{T_{\theta_N}} \boldsymbol{R_{\phi_{n-1}}} \boldsymbol{T_{\theta_{C^\alpha}}} \begin{bmatrix} d_{C^\alpha} \\ 0 \\ 0 \end{bmatrix} + \boldsymbol{T_{\theta_{C'}}} \boldsymbol{R_\omega} \boldsymbol{T_{\theta_N}} \begin{bmatrix} d_N \\ 0 \\ 0 \end{bmatrix} + \boldsymbol{T_{\theta_{C'}}} \begin{bmatrix} d_{C'} \\ 0 \\ 0 \end{bmatrix} + \begin{bmatrix} d_{C^\alpha} \\ 0 \\ 0 \end{bmatrix}. \end{aligned}$$

Let matrix $\boldsymbol{M_i} = \boldsymbol{T_{\theta_{C'}}} \boldsymbol{R_\omega} \boldsymbol{T_{\theta_N}} \boldsymbol{R_{\phi_i}} \boldsymbol{T_{\theta_{C^\alpha}}} \boldsymbol{R_{\psi_i}}$ and $\boldsymbol{q} = \boldsymbol{T_{\theta_{C'}}} \boldsymbol{R_\omega} \boldsymbol{T_{\theta_N}} \begin{bmatrix} d_N \\ 0 \\ 0 \end{bmatrix} + \boldsymbol{T_{\theta_{C'}}} \begin{bmatrix} d_{C'} \\ 0 \\ 0 \end{bmatrix} + \begin{bmatrix} d_{C^\alpha} \\ 0 \\ 0 \end{bmatrix}$, then

$$\boldsymbol{r_{3n-2}} = \boldsymbol{q}, \quad \boldsymbol{r_{3n-5}} = \boldsymbol{M_{n-1}} \boldsymbol{q} + \boldsymbol{q}.$$

If we continue the process, we obtain

$$\begin{aligned} \boldsymbol{r_{3n-8}} &= \boldsymbol{M_{n-2}} \boldsymbol{M_{n-1}} \boldsymbol{q} + \boldsymbol{M_{n-2}} \boldsymbol{q} + \boldsymbol{q}, \\ \boldsymbol{r_{3n-11}} &= \boldsymbol{M_{n-3}} \boldsymbol{M_{n-2}} \boldsymbol{M_{n-1}} \boldsymbol{q} + \boldsymbol{M_{n-3}} \boldsymbol{M_{n-2}} \boldsymbol{q} + \boldsymbol{M_{n-3}} \boldsymbol{q} + \boldsymbol{q}, \quad \cdots \end{aligned}$$

Finally, the requirement for having the same origin is

$$\boldsymbol{r_1} = \boldsymbol{M_1} \boldsymbol{M_2} \cdots \boldsymbol{M_{n-2}} \boldsymbol{M_{n-1}} \boldsymbol{q} + \boldsymbol{M_1} \boldsymbol{M_2} \cdots \boldsymbol{M_{n-2}} \boldsymbol{q} + \cdots + \boldsymbol{M_1} \boldsymbol{M_2} \boldsymbol{q} + \boldsymbol{M_1} \boldsymbol{q} + \boldsymbol{q} = \boldsymbol{0}. \quad (5)$$

For vectors, the translation $\boldsymbol{p_i}$ in Eq (1) can be ignored. Going through the same derivation process, a vector $\boldsymbol{v_{3n+1}}$ in system $3n+1$ has its vector form

$$\boldsymbol{v_1} = \boldsymbol{M_1} \boldsymbol{M_2} \cdots \boldsymbol{M_{n-2}} \boldsymbol{M_{n-1}} \boldsymbol{M_n} \boldsymbol{v_{3n+1}}$$

in system 1. Therefore, the $\boldsymbol{x}$ and $\boldsymbol{y}$ directional vectors in system $3n+1$ have vector forms

$$\boldsymbol{M_1} \boldsymbol{M_2} \cdots \boldsymbol{M_{n-2}} \boldsymbol{M_{n-1}} \boldsymbol{M_n} \boldsymbol{e_1}, \quad \boldsymbol{M_1} \boldsymbol{M_2} \cdots \boldsymbol{M_{n-2}} \boldsymbol{M_{n-1}} \boldsymbol{M_n} \boldsymbol{e_2},$$

in system 1, respectively, with

$$\boldsymbol{e_1} = \begin{bmatrix} 1 \\ 0 \\ 0 \end{bmatrix}, \quad \boldsymbol{e_2} = \begin{bmatrix} 0 \\ 1 \\ 0 \end{bmatrix}.$$
