# Supplementary material for: Heuristic energy-based cyclic peptide design
Source: PLoS Comput Biol. 2025 Apr 30;21(4):e1012290. doi: 10.1371/journal.pcbi.1012290 (PMC12043242; doi:10.1371/journal.pcbi.1012290)
Supplement: S2 Text — (PDF) [file pcbi.1012290.s002.pdf]

2 Backbone energy functions

We choose Rosetta's *Ref2015* energy model.[1] We compute energies for the backbone atoms, which are Ramachandran energy, repulsive energy, attractive energy, electrostatic energy, solvation energy, and hydrogen bond energy.

As all backbone residues are assumed to be glycine, we use the permissive, flattened glycine Ramachandran space to calculate backbone Ramachandran energy. We have the Ramachandran energy on the corners of grid cells based on a 10° spacing. For any point inside a grid cell, we calculate its Ramachandran energy by bicubic interpolation of the values on the cell corners. In particular, for a cell with lower left corner at (ϕ_m, ψ_n), its bicubic interpolation is

$$E_{rama}^{(m,n)}(\phi, \psi) = \sum_{i=0}^{3} \sum_{j=0}^{3} a_{ij}^{(m,n)} \left(\frac{\phi - \phi_m}{\Delta\phi} \right)^i \left(\frac{\psi - \psi_n}{\Delta\psi} \right)^j,$$

where $\Delta\phi = \Delta\psi = 10°$. The coefficients $a_{ij}^{(m,n)}$ are set to ensure continuous energy values E_{rama} and partial derivatives $\frac{\partial E_{rama}}{\partial \phi}$, $\frac{\partial E_{rama}}{\partial \psi}$, and $\frac{\partial^2 E_{rama}}{\partial \phi \partial \psi}$ at the four corners. We compute and save these bicubic interpolation coefficients in advance for all cells.

When analyzing backbone atom pair interactions, we follow Rosetta's *Ref2015* energy model.[1] In this model, only atom pairs separated by at least 4 covalent bonds are considered in order to prevent dominance by short-range interactions. We represent backbone atoms as nodes and covalent bonds as edges of length one. By calculating the shortest paths between each atom pair, we compute energies for pairs with path lengths ≥ 4. This approach aligns with the rationale in the Rosetta paper[1] to "exclude the large repulsive energetic contributions that would otherwise be calculated for atoms separated by fewer than four chemical bonds". For *Van der Waals* interactions, we follow the *Ref2015* model to split the Lennard-Jones 6-12 potential into repulsive and attractive energies. Similarly, we approximate Coulomb's law to compute electrostatic energies, and apply the Lazaridis-Karplus implicit Gaussian exclusion model to compute isotropic solvation energies. Example energy plots are shown in Fig. S1a for the backbone atom pair N and C′.

Hydrogen bond energy consists of three components E_{hbond}^{HA}, E_{hbond}^{AHD}, and $E_{hbond}^{B_2BAH}$. H refers to the hydrogen atom, and D is its donor (backbone atom N, Fig. S1b). A refers to the acceptor (atom O), B its base (atom C′), and $B2$ is the parent (atom C^α). The distance between the hydrogen and its acceptor is d_{HA}. The bond angle between A, H, and D is θ_{AHD}. The bond angle between B, A, and H is θ_{BAH}. The torsion angle between $B2$, B, A, and H is χ_{B2BAH}. If the bond angles satisy $90° \leq \theta_{AHD}$ and $\theta_{BAH} \leq 180°$, then the energy component E_{hbond}^{HA} forms the curve shown in Fig. S1b; otherwise, $E_{hbond}^{HA} = 0$. Similarly, if $d_{HA} \leq 3.2$ Å and $90° \leq \theta_{BAH} \leq 180°$, then the energy component E_{hbond}^{AHD} forms the curve in Fig. S1b; otherwise, $E_{hbond}^{AHD} = 0$. We draw the Lambert azimuthal project of $E_{hbond}^{B_2BAH}$ in Fig. S1b, with θ_{BAH} corresponding to the radius and χ_{B2BAH} rotating counterclockwise. The final hydrogen bond energy $E_{hbond} = w_H w_A f(E_{hbond}^{HA} +$

$E_{hbond}^{AHD} + E_{hbond}^{B_2BAH}$), where $w_H = 1.41$, $w_A = 1.08$ for backbone hydrogen bond, and

$$f(x) = \begin{cases} x & \text{if } x < -0.1 \\ -0.025 + \frac{x}{2} - 2.5x^2 & \text{if } -0.1 \leq x < 0.1 \\ 0 & \text{if } 0.1 \leq x \end{cases}$$

We consider a hydrogen bond to be formed if $E_{hbond} < -0.25$.

References

[1] R. Alford, A. Leaver-Fay, J. Jeliazkov, M. O'Meara, et al. The rosetta all-atom energy function for macromolecular modeling and design. *J. Chem. Theory Comput.*, 13:3031–3048, 2017.
