# Supplementary material for: Heuristic energy-based cyclic peptide design
Source: PLoS Comput Biol. 2025 Apr 30;21(4):e1012290. doi: 10.1371/journal.pcbi.1012290 (PMC12043242; doi:10.1371/journal.pcbi.1012290)
Supplement: S3 Text — (PDF) [file pcbi.1012290.s003.pdf]

3 Layered simulated annealing

To build initial backbone configurations, we partition the glycine Ramachandran map into six torsion bins (Figure S2). For each backbone residue, its initial angles ϕ and ψ are chosen randomly from one of the six torsion bin centers. Figure S2 shows an example 7-residue polyglycine chain, with all initial angles chosen as center 1.

For each initial backbone configuration, we perform the layered simulated annealing. Each layer contains a Metropolis criterion to decide whether the new configuration should be accepted or not. In the cyclic error Metropolis criterion, the energy threshold $E_{thr,cyc}$ is set to be 0.3 for 7 and 15 residues, so that the N-C terminal bond angles and bond lengths deviate little from the ideal values. For 20 and 24 residues, we choose a more lenient $E_{thr,cyc}$ threshold of 1, to find enough backbone candidates under the strict hydrogen bond requirements. Though the N-C terminal bond angles and lengths deviate more, they are still within reasonable ranges to form the peptide bond, and can easily be relaxed to near-ideal geometry by the subsequent application of the Rosetta FastRelax protocol.[1]

For Ramachandran energy, the thresholds are $8n$, where n is the number of residues in the backbone; for hydrogen bonding, we use H-bond count instead of the exact H-bond energy, and the count thresholds are $\lceil n/3 \rceil$ (Table S1). The repulsive and miscellaneous energy thresholds are tuned for a ~15% configuration acceptance rate.

We consider a configuration to be a good backbone candidate, if its repulsive energy $\leq E_{cri,rep}$, cyclic error $\leq E_{cri,cyc}$, and hydsrogen bonds $\geq H_{cri,count}$ (Table S1). We show an example 7-residue good candidate with energies labeled in Fig S2. To design cyclic peptides of intermediate sizes in the future, these thresholds and criteria can be determined using linear interpolation.

A total of M=10000 time steps are performed in the layered simulated annealing, and the random move disk radius k_t and temperatures $T_{t,l}$ decrease as a function of time as follows:

$$k_t = \frac{k_0}{1 + b * t/M}, \quad T_{t,l} = \frac{T_{0,l}}{1 + c_l * t/M}.$$

The initial temperature parameters $T_{0,l}$ are chosen to be two times the average energy deviations in test runs, to generate an initial passing probability of ~ 0.6 (Table S1). Since these $T_{0,l}$ values exhibit a linear growth pattern, linear interpolation can again be used to determine their values for future cyclic peptide design.

For the other simulated annealing parameters (initial random move disk radius k_0, disk shrinking rate b, and temperature dropping rates c_{rama}, c_{rep}, c_{cyc}, c_{hbond}, and c_{other}), approximate ranges are found in test runs and are divided evenly between 3 and 8 values. Within the thousands of possible simulated annealing parameter combinations, we use combinatorial design to select 51 combinations for 7- and 15-residue tests, and 400 combinations for 20- and 24-residue tests (see **??**).

The pseudocode for our layered simulated annealing algorithm is provided below.

Algorithm 1 Pseudocode for layered simulated annealing

Input: (i) Peptide sequence (all glycine for backbone sampling); (ii) Number of initial points N_p; (iii) Simulated annealing parameters M, $E_{thr,rama}$, $E_{thr,rep}$, $E_{thr,cyc}$, $H_{thr,count}$, $E_{thr,other}$ (optional), $E_{cri,rep}$, $E_{cri,cyc}$, $H_{cri,count}$, k_0, b, $T_{0,rama}$, $T_{0,rep}$, $T_{0,cyc}$, $T_{0,hbond}$, $T_{0,other}$ (optional), c_{rama}, c_{rep}, c_{cyc}, c_{hbond}, c_{other} (optional).

Output: Good backbone candidates and their energies E_{rama}, E_{rep}, E_{hbond}, E_{other} (optional).

```
Randomly select N_p initial points from possible combinations of torsion bin centers
for each initial point angles do
    Calculate initial energies E_rama, E_rep, E_cyc, E_hbond, and E_other, and H-bound count H_count
    N_repeat ← 1, N_backbone ← 0
    while N_repeat ≤ 3 and N_backbone = 0 do
        for time step t from 1 to M do
            k_t ← k_0 / (1+b*t/M)
            Generate a random move within a disk of radius k_t for each residue
            Record the new point angles_new generated

            Calculate Ramachandran energy E_new,rama at the new point        ▷ Rama energy test
            rama_explore ← False, T_t,rama ← T_0,rama / (1+c_rama*t/M)
            if E_new,rama ≤ E_rama or E_new,rama ≤ E_thr,rama then
                rama_explore ← True
            else
                With probability e^((E_rama − E_new,rama)/T_t,rama), set rama_explore True
            end if                                                           ▷ Rama energy test ends

            if rama_explore is True then                                     ▷ Repulsive energy test
                Calculate repulsive energy E_new,rep at the new point
                rep_explore ← False, T_t,rep ← T_0,rep / (1+c_rep*t/M)
                if E_new,rep ≤ E_rep or E_new,rep ≤ E_thr,rep then
                    rep_explore ← True
                else
                    With probability e^((E_rep − E_new,rep)/T_t,rep), set rep_explore True
                end if                               ▷ Metropolis criterion for repulsive energy ends

                if rep_explore is True then                                  ▷ Cyclic error test
                    Calculate cyclic error E_new,cyc at the new point
                    cyc_explore ← False, T_t,cyc ← T_0,cyc / (1+c_cyc*t/M)
```

if $E_{new,cyc} \leq E_{cyc}$ **or** $E_{new,cyc} \leq E_{thr,cyc}$ **then**
    cyc_explore $\leftarrow$ True
else
    With probability $e^{(E_{cyc}-E_{new,cyc})/T_{t,cyc}}$, set *cyc_explore* True
end if ▷ Metropolis criterion for cyclic error ends

if *cyc_explore* is True **then** ▷ Hydrogen bond energy test
    Calculate hydrogen bond energy $E_{new,hbond}$ at the new point
    hbond_explore $\leftarrow$ False, $T_{t,hbond} \leftarrow \frac{T_{0,hbond}}{1+c_{hbond}*t/M}$
    if $E_{new,hbond} \leq E_{hbond}$ **or** $H_{new,count} \geq H_{thr,count}$ **then**
        hbond_explore $\leftarrow$ True
    else
        With probability $e^{(E_{hbond}-E_{new,hbond})/T_{t,hbond}}$, set *hbond_explore* True
    end if ▷ Metropolis criterion for hydrogen bond energy ends

    if *hbond_explore* is True **then** ▷ Optional miscellaneous energy test
        Calculate miscellaneous energy $E_{new,other}$ at the new point
        accept $\leftarrow$ False, $T_{t,other} \leftarrow \frac{T_{0,other}}{1+c_{other}*t/M}$
        if $E_{new,other} \leq E_{other}$ **or** $E_{new,other} \leq E_{thr,other}$ **then**
            accept $\leftarrow$ True
        else
            With probability $e^{(E_{other}-E_{new,other})/T_{t,other}}$, set *accept* True
        end if ▷ Metropolis criterion for miscellaneous energy ends

        if *accept* is True **then** ▷ Accept the new point
            $angles \leftarrow angles\_new$, $E_{rama} \leftarrow E_{new,rama}$, $E_{rep} \leftarrow E_{new,rep}$,
            $E_{cyc} \leftarrow E_{new,cyc}$, $E_{hbond} \leftarrow E_{new,hbond}$, $H_{count} \leftarrow H_{new,count}$,
            $E_{other} \leftarrow E_{new,other}$
            if $E_{rep} \leq E_{cri,rep}$ **and** $E_{cyc} \leq E_{cri,cyc}$ **and** $H_{count} \geq H_{cri,count}$ **then**
                Record *angles* and its energies as a good backbone candidate
                $N_{backbone} \leftarrow N_{backbone} + 1$
            end if ▷ The backbone is recorded as a candidate
        end if ▷ The new point is accepted
    end if ▷ Miscellaneous energy test ends
end if ▷ Hydrogen bond energy test ends
end if ▷ Cyclic error test ends
end if ▷ Repulsive energy test ends
end for
$N_{repeat} \leftarrow N_{repeat} + 1$
end while
end for

References

[1] F. Khatib, S. Cooper, M. Tyka, K. Xu, et al. Algorithm discovery by protein folding game players. *Proc. Natl. Acad. Sci. U.S.A.*, 108:18949–18953, 2011.
