# Supplementary material for: Heuristic energy-based cyclic peptide design
Source: PLoS Comput Biol. 2025 Apr 30;21(4):e1012290. doi: 10.1371/journal.pcbi.1012290 (PMC12043242; doi:10.1371/journal.pcbi.1012290)
Supplement: S4 Text — (PDF) [file pcbi.1012290.s004.pdf]

4 Combinatorial design

The goal of combinatorial design is to obtain well-spaced random samples from the cross-product of parameter-values.[1] In our case, we are performing a simulated annealing parameter choice, as described in Layered simulated annealing. Specifically, our combinatorial design method guarantees that for any pair of parameters P1 and P2, every possible combination of the discrete values of P1 and P2, such as P1.v1 and P2.v2, is present in at least one initial parameter setting. Note that this differs from exhaustive search because, for example, P1.v1 and P2.v2 may be in the same initial setting as some values P3.v3 and P4.v4, but never in the same initial setting as some other values P3.v3' and P4.v4'.

While an exhaustive search would require 61440 parameter settings for 7 residue backbone sampling (7 parameters k_0, b, c_{rama}, c_{rep}, c_{cyc}, c_{hbond}, and c_{other}, each between 3 and 8 values), this form of combinatorial design requires only 51 settings. The same settings are used for the 15 residue simulated annealing parameter optimization.

For 20 and 24 residues, we employ a special form of combinatorial design that has pivots. It uses the generic combinatorial design to find settings of the non-pivot parameters, and repeats the settings for every value of the pivot parameters. We choose initial random move disk radius k_0 and disk shrinking rate b as pivots, because, in test runs, they have a large impact on successfully finding good backbones that satisfy the repulsive energy, cyclic error, and hydrogen bond requirements. Parameter k_0 has four discrete values (0.6, 0.7, 0.8, and 0.9), b has five values (15, 16, 17, 18, and 19), and the other parameters yield 20 settings by the generic combinatorial design. As a result, the pivot combinatorial design generates 400 ($20 \times 4 \times 5$) parameter settings.

For each parameter combination, after setting the initial angle as center 1 for all residues (Fig 3a of main text), we repeat the layered simulated annealing 20 times for 7 and 15 residues, and 10 times for 20 and 24 residues. In each run of simulated annealing, we record the number of good candidate backbones produced (satisfying criteria in Table S1). The optimal parameter combination is the one which produces candidate backbones in more than half of the simulated annealing runs and has the most candidates in total. For future design of other intermediate sizes, similar parameter optimization can be performed, and we suggest parameter ranges of 0.6-0.9 for k_0, 15-19 for b, 2-4 for c_{rama}, 12-18 for c_{rep}, 14-20 for c_{cyc}, 16-22 for c_{hbond}, and 4-10 for c_{other}.

Simulations were performed on the Greene supercomputer clusters at the New York University's High Performance Computing facilities. Each compute node in the Greene clusters has two 24-core Intel Cascade Lake Platinum 8268 chips and 192 GB memory. The parameter combination selection process took 9.6, 16, 33.6, and 41.6 CPU hours for 7, 15, 20, and 24 residues, respectively.

References

[1] C. Colbourn, S. Martirosyan, G. Mullen, D. Shasha, G. Sherwood, and J. Yucas. Products of mixed covering arrays of strength two. *J. Combin. Designs*, 14:124–138, 2006.
