# Supplementary material for: Heuristic energy-based cyclic peptide design
Source: PLoS Comput Biol. 2025 Apr 30;21(4):e1012290. doi: 10.1371/journal.pcbi.1012290 (PMC12043242; doi:10.1371/journal.pcbi.1012290)
Supplement: S5 Text — (PDF) [file pcbi.1012290.s005.pdf]

5 Torsion angle FastRelax script

```
<ROSETTASCRIPTS>
	# The SCOREFXNS section defines scoring functions
	<SCOREFXNS>
		# The current Rosetta default scorefunction
		<ScoreFunction name="ref" weights="ref2015" />
		# Use chainbreak to maintain cyclic
		<ScoreFunction name="ref_chainbreak" weights="ref2015" >
			<Reweight scoretype="chainbreak" weight="15.0" />
		</ScoreFunction>
	</SCOREFXNS>
	# The SIMPLE_METRICS section allows users to configure metrics used to measure
    properties of a structure.
	<SIMPLE_METRICS>
		# Metric to measure backbone hydrogen bonds
		<PeptideInternalHbondsMetric name="internal_hbonds" />
		# Metric to measure score/energy
		<TotalEnergyMetric name="score" scorefxn="ref" />
	</SIMPLE_METRICS>
	# The FILTERS section allows users to configure filters. These measure
    properties of a structure and make decisions, based on the measured properties,
    about whether to discard the current structure.
	<FILTERS>
		# Filter to avoid score function artifact of having more than two
    hydrogen bonds to carbonyls
		<OversaturatedHbondAcceptorFilter name="oversat" scorefxn="ref"
    max_allowed_oversaturated="0" consider_mainchain_only="false"/>
		# Filter to ensure a minimum number of hbonds (peptide size n/3)
		<PeptideInternalHbondsFilter name="min_internal_hbonds" hbond_cutoff="7"
    />
	</FILTERS>
	# The MOVERS section allows users to define movers, which are Rosetta modules
    that modify a structure in some way.
	<MOVERS>
		# A mover to declare a bond connecting the termini (i.e. to cyclize the
    peptide). Note that the user needs to input variable %%Nres%% (peptide size) when
    running this relaxation script. Use option "-parser:script_vars Nres=20" for 20
    residue relaxation.
		<DeclareBond name="peptide_bond1" res1="1" atom1="N" atom2="C"
    res2="%%Nres%%" add_termini="true" />
		# Three repeats of fastrelax
		<FastRelax name="frlx" scorefxn="ref_chainbreak" repeats="3"
    ramp_down_constraints="false" >
			# All side-chain and backbone torsion angles can move
```

```
                        <MoveMap name="frlx_mm" >
                                <Chain number="1" chi="true" bb="true" />
                        </MoveMap>
                </FastRelax>
                # These movers allow the simple metrics to be run
                <RunSimpleMetrics name="measure_internal_hbonds"
    metrics="internal_hbonds" />
                <RunSimpleMetrics name="measure_score" metrics="score" />
        </MOVERS>
        # The PROTOCOLS section is the section in which the user invokes the modules
    defined above in linear sequence to define a protocol:
        <PROTOCOLS>
                <Add mover="peptide_bond1" />
                <Add mover="frlx" />
                # A side-effect of the DeclareBond mover is the correction of positions
    of H and O atoms that depend on the peptide bond. We re-invoke it here for that
    purpose.
                <Add mover="peptide_bond1" />
                <Add filter="min_internal_hbonds" />
                <Add filter="oversat" />
        </PROTOCOLS>
        # The OUTPUT section allows the user to define output settings. Here, we specify
    the scoring function that will be used to score the output structure.
        <OUTPUT scorefxn="ref"/>
</ROSETTASCRIPTS>
```
