# Supplementary material for: Heuristic energy-based cyclic peptide design
Source: PLoS Comput Biol. 2025 Apr 30;21(4):e1012290. doi: 10.1371/journal.pcbi.1012290 (PMC12043242; doi:10.1371/journal.pcbi.1012290)
Supplement: S6 Text — (PDF) [file pcbi.1012290.s006.pdf]

6 Small macrocycle FastDesign script

```
<ROSETTASCRIPTS>
	<SCOREFXNS>
		<ScoreFunction name="ref" weights="ref2015" />
		# The default scorefunction with increased hydrogen bond weights, and with aa_composition, aspartimide_penalty, and chainbreak scores activated.
		<ScoreFunction name="ref_highhbond" weights="ref2015" >
			<Reweight scoretype="hbond_lr_bb" weight="5.0" />
			<Reweight scoretype="hbond_sr_bb" weight="5.0" />
			<Reweight scoretype="aa_composition" weight="1.0" />
			<Reweight scoretype="aspartimide_penalty" weight="1.0" />
			<Reweight scoretype="chainbreak" weight="15.0" />
		</ScoreFunction>
	</SCOREFXNS>
	# The PACKER_PALETTES section defines the residues available for design.
	<PACKER_PALETTES>
		<CustomBaseTypePackerPalette name="palette" additional_residue_types
="DALA,DASP,DGLU,DPHE,DHIS,DILE,DLYS,DLEU,DMET,DASN,DPRO,DGLN,DARG,DSER,DTHR,
DVAL,DTRP,DTYR" />
	</PACKER_PALETTES>
	# The RESIDUE_SELECTORS section allows users to select residues.
	<RESIDUE_SELECTORS>
		# Select residues with mainchain phi torsion values greater than zero. These positions will be restricted to becoming D-amino acids during design.
		<Phi name="posPhi" select_positive_phi="true" />
		# Select residues with mainchain phi torsion values less than zero. These positions will be restricted to becoming L-amino acids during design.
		<Phi name="negPhi" select_positive_phi="false" />
	</RESIDUE_SELECTORS>
	<SIMPLE_METRICS>
		<PeptideInternalHbondsMetric name="internal_hbonds" />
	</SIMPLE_METRICS>
	<FILTERS>
		<OversaturatedHbondAcceptorFilter name="oversat" scorefxn="ref" max_allowed_oversaturated="0" consider_mainchain_only="false"/>
		<PeptideInternalHbondsFilter name="min_internal_hbonds" hbond_cutoff="5" />
	</FILTERS>
	# The TASKOPERATIONS section allows users to control side-chain identity.
	<TASKOPERATIONS>
		# Task operation to read a resfile defining the D-amino acids, used for design at positions with mainchain phi torsion values greater than zero.
		<ReadResfile name="d_res" filename="d_res.txt" selector="posPhi"/>
		# Task operation to read a resfile defining the L-amino acids, used for
```

```
    design at positions with mainchain phi torsion values less than zero.
            <ReadResfile name="l_res" filename="l_res.txt" selector="negPhi"/>
        </TASKOPERATIONS>
        <MOVERS>
            <DeclareBond name="peptide_bond1" res1="1" atom1="N" atom2="C"
    res2="%%Nres%%" add_termini="true" />
            # Composition constraints add a nonlinearly-ramping penalty for
    deviation from a desired amino acid composition written in the .comp file.
            <AddCompositionConstraintMover name="addcompcsts"
    filename="desired_makeup.comp" />
            # The FastDesign mover performs alternating rounds of sequence design
    and torsion-space energy minimization, while ramping the repulsive term in the
    scorefunction (fa_rep).
            <FastDesign name="fdes" scorefxn="ref_highhbond" repeats="3"
    task_operations="d_res,l_res" packer_palette="palette" ramp_down_constraints="false"
    >
                <MoveMap name="fdes_mm" >
                    <Chain number="1" chi="true" bb="true" />
                </MoveMap>
            </FastDesign>
            <RunSimpleMetrics name="measure_internal_hbonds"
    metrics="internal_hbonds" />
        </MOVERS>
        <PROTOCOLS>
            <Add mover="peptide_bond1" />
            <Add mover="addcompcsts" />
            <Add mover="fdes" />
            <Add mover="peptide_bond1" />
            <Add filter="oversat" />
            <Add filter="min_internal_hbonds" />
        </PROTOCOLS>
        <OUTPUT scorefxn="ref"/>
</ROSETTASCRIPTS>
```

l_res.txt

```
PIKAA ADEFHIKLMNPQRSTVWY
start
```

d_res.txt

```
PIKAA X[DALA]X[DASP]X[DGLU]X[DPHE]X[DHIS]X[DILE]X[DLYS]X[DLEU]X[DMET]X[DASN]X[DPRO]
X[DGLN]X[DARG]X[DSER]X[DTHR]X[DVAL]X[DTRP]X[DTYR]
```

desired_makeup.comp

```
# At least two proline residues. These can be L- or D- (or mixed).
PENALTY_DEFINITION
TYPE PRO DPR
DELTA_START -2
DELTA_END 1
PENALTIES 500 10 0 0
ABSOLUTE 2
BEFORE_FUNCTION QUADRATIC
AFTER_FUNCTION CONSTANT
END_PENALTY_DEFINITION

# At least one L-asp or L-glu.
PENALTY_DEFINITION
TYPE ASP GLU
DELTA_START -1
DELTA_END 1
PENALTIES 200 0 0
ABSOLUTE 1
BEFORE_FUNCTION QUADRATIC
AFTER_FUNCTION CONSTANT
END_PENALTY_DEFINITION

# At least one positively-charged residue
PENALTY_DEFINITION
TYPE LYS ARG DLY DAR
DELTA_START -1
DELTA_END 1
PENALTIES 200 0 0
ABSOLUTE 1
BEFORE_FUNCTION QUADRATIC
AFTER_FUNCTION CONSTANT
END_PENALTY_DEFINITION
```
