# Supplementary material for: Heuristic energy-based cyclic peptide design
Source: PLoS Comput Biol. 2025 Apr 30;21(4):e1012290. doi: 10.1371/journal.pcbi.1012290 (PMC12043242; doi:10.1371/journal.pcbi.1012290)
Supplement: S7 Text — (PDF) [file pcbi.1012290.s007.pdf]

7 Large macrocycle FastDesign script

```
<ROSETTASCRIPTS>
	<SCOREFXNS>
		<ScoreFunction name="ref" weights="ref2015" />
		<ScoreFunction name="ref_highhbond" weights="ref2015" >
			<Reweight scoretype="hbond_lr_bb" weight="10.0" />
			<Reweight scoretype="hbond_sr_bb" weight="10.0" />
			<Reweight scoretype="aa_composition" weight="1.0" />
			<Reweight scoretype="aspartimide_penalty" weight="1.0" />
			<Reweight scoretype="chainbreak" weight="25.0" />
		</ScoreFunction>
	</SCOREFXNS>
	<PACKER_PALETTES>
		<CustomBaseTypePackerPalette name="palette" additional_residue_types
="DALA,DASP,DGLU,DPHE,DHIS,DILE,DLYS,DLEU,DMET,DASN,DPRO,DGLN,DARG,DSER,DTHR,
DVAL,DTRP,DTYR" />
	</PACKER_PALETTES>
	<RESIDUE_SELECTORS>
		<Phi name="posPhi" select_positive_phi="true" />
		<Phi name="negPhi" select_positive_phi="false" />
		# Select the most buried residues
		<Layer name="select_core" select_core="true" select_boundary="false"
select_surface="false" core_cutoff="2.5" surface_cutoff="1.0" />
		# Select partially buried residues
		<Layer name="select_boundary" select_core="false" select_boundary="true"
select_surface="false" core_cutoff="2.5" surface_cutoff="1.0" />
		# Select fully solvent-exposed residues
		<Layer name="select_surface" select_core="false" select_boundary="false"
select_surface="true" core_cutoff="2.5" surface_cutoff="1.0" />
	</RESIDUE_SELECTORS>
	<SIMPLE_METRICS>
		<PeptideInternalHbondsMetric name="internal_hbonds" />
	</SIMPLE_METRICS>
	<FILTERS>
		<OversaturatedHbondAcceptorFilter name="oversat" scorefxn="ref"
max_allowed_oversaturated="0" consider_mainchain_only="false"/>
		<PeptideInternalHbondsFilter name="min_internal_hbonds" hbond_cutoff="7"
/>
	</FILTERS>
	<TASKOPERATIONS>
		<ReadResfile name="d_res" filename="d_res.txt" selector="posPhi"/>
		<ReadResfile name="l_res" filename="l_res.txt" selector="negPhi"/>
		# At buried positions, restrict to PFAMILYVW and D-aa equivalents
		<RestrictToSpecifiedBaseResidueTypes name="core_restrictions"
```

```
    base_types="PRO,PHE,ALA,MET,ILE,LEU,TYR,VAL,TRP,DPRO,DPHE,DALA,DMET,DILE,DLEU,
    DTYR,DVAL,DTRP" selector="select_core"/>
            # At semi-buried positions, PAILYVNQDERKSTH and D-aa equivalents
            <RestrictToSpecifiedBaseResidueTypes name="boundary_restrictions"
    base_types="PRO,ALA,ILE,LEU,TYR,VAL,ASN,GLN,ASP,GLU,ARG,LYS,SER,THR,HIS,DPRO,
    DALA,DILE,DLEU,DTYR,DVAL,DASN,DGLN,DASP,DGLU,DARG,DLYS,DSER,DTHR,DHIS"
    selector="select_boundary"/>
            # At surface-exposed positions, PANQDERKSTH and D-aa equivalents
            <RestrictToSpecifiedBaseResidueTypes name="surf_restrictions"
    base_types="PRO,ALA,ASN,GLN,ASP,GLU,ARG,LYS,SER,THR,HIS,DPRO,DALA,DASN,DGLN,
    DASP,DGLU,DARG,DLYS,DSER,DTHR,DHIS" selector="select_surface"/>
        </TASKOPERATIONS>
        <MOVERS>
            <DeclareBond name="peptide_bond1" res1="1" atom1="N" atom2="C"
    res2="%%Nres%%" add_termini="true" />
            <AddCompositionConstraintMover name="addcompcsts"
    filename="desired_makeup.comp" />
            <FastDesign name="fdes" scorefxn="ref_highhbond" repeats="3"
    task_operations="d_res, l_res, core_restrictions, boundary_restrictions,
    surf_restrictions" packer_palette="palette" ramp_down_constraints="false" >
                <MoveMap name="fdes_mm" >
                    <Chain number="1" chi="true" bb="true" />
                </MoveMap>
            </FastDesign>
            <RunSimpleMetrics name="measure_internal_hbonds"
    metrics="internal_hbonds" />
        </MOVERS>
        <PROTOCOLS>
            <Add mover="peptide_bond1" />
            <Add mover="addcompcsts" />
            <Add mover="fdes" />
            <Add mover="peptide_bond1" />
            <Add filter="oversat" />
            <Add filter="min_internal_hbonds" />
        </PROTOCOLS>
        <OUTPUT scorefxn="ref"/>
</ROSETTASCRIPTS>
```
