# Supplementary material for: Heuristic energy-based cyclic peptide design
Source: PLoS Comput Biol. 2025 Apr 30;21(4):e1012290. doi: 10.1371/journal.pcbi.1012290 (PMC12043242; doi:10.1371/journal.pcbi.1012290)
Supplement: S8 Text — (PDF) [file pcbi.1012290.s008.pdf]

8 ClusterGen stability analysis

For macrocycles having 15-24 residues, the Ramachandran-stability filtering method fails to find sufficient alternative backbone conformations due to the exponentially larger search space. There are six different types of Ramachandran spaces (see Fig. S3). The L amino acid Ramachandran spaces hardly overlap with those of the D amino acids, so the chance that a residue's torsion angles fit into the corresponding Ramachandran space is roughly 1/2. This gives a probability of $\sim \frac{1}{2^{15}/15}$ or $\frac{1}{2^{24}/24}$ of finding a compatible backbone candidate with a 15- or 24-residue sequence, respectively. Therefore, efficient search of low-energy alternative conformations is needed.

To form the initial population of ClusterGen, we perform two separate layered simulated annealing, one for low energy, and one for low RMSD. The low energy simulated annealing is the same as in Layered simulated annealing, while the low RMSD simulated annealing has only the cyclic error test and a RMSD test (see pseudocode below). All parameters follow Table 1 of main text, and the newly added RMSD parameters are threshold $E_{thr,rmsd}$=1.5 Å, the 15- and 20-residue criterion $E_{cri,rmsd}$=1.5 Å, the 24-residue criterion $E_{cri,rmsd}$=1.7 Å, the initial temperature $T_{0,rmsd}$=1, and the temperature dropping rate c_{rmsd}=50, chosen by test runs. For both low energy and low RMSD simulated annealing procedures of 15 residues, we randomly select 1000 initial points from possible combinations of torsion bin centers corresponding to the designed sequence (Fig. S3). For 20 residues, we select 10000 initial points for each SA procedure. For 24 residues, we select 20000 initial points for low energy SA, and 40000 for low RMSD SA.

The sampled backbones are then subject to FastRelax. We sort the relaxed structures in ascending order of their energies and initiate energy-based clustering. Each time, we select the lowest-energy structure from the remaining unclustered structures (the first one in the sorted list) to serve as a new cluster center. Then, we measure the backbone-heavy-atom RMSDs of the other unclustered structures from this new center. We assign those having RMSDs smaller than 0.5 Å to this cluster, and remove them from the sorted list. We repeat this process until all structures have been assigned to clusters. Note that we record only coordinates of backbone heavy atoms during the genetic algorithm to save memory and computation time, but we relax entire structures including sidechains during FastRelax to obtain full energies.

In the genetic algorithm, each generation undergoes crossover, mutation, and selection (see pseudocode below). Crossover involves checking if a pair of parents can exchange residues within a designated region. More specifically, for two breakpoint residues $b1$ and $b2$ (i.e., exchange residues $b1+1$ to $b2$), we use the Kabsch algorithm[1] to align eight atoms of the two parents: C^{α} and C' of residues $b1$ and $b2$, atoms N and C^{α} of residues $b1+1$ and $b2+1$ (see Fig. S4). The algorithm first translates both sets of atoms to have their centroids at the origin. Then, it computes the covariance matrix between the two sets of atoms and performs singular value decomposition on this matrix to determine the optimal rotation matrix. This rotation minimizes the sum of the squared distances between corresponding atoms, and the root mean of this sum is the RMSD. If the alignment yields an RMSD $\leq rsmd_{xover}$ (0.5 Å) per atom, the parents exchange these residues to generate two

children.

Given a mutation region spanning residues $d1$ to $d2$, we add a random perturbation of up to $p_{max} = 10^\circ$ to the torsion angles of residue $d1$. Subsequently, we adjust the torsion angles of the remaining residues to ensure a smooth connection with residue $d2 + 1$ (see Fig. S4). Specifically, we employ a simulated annealing approach with $M_{mut} = 1000$ steps. In each step t, we add new perturbations of $\leq p_t$ to the current torsion angles of residues $d1 + 1$ to $d2$, where $p_t = \frac{10}{1+9*t/1000}$. If a perturbation steps out of the associated Ramachandran space, it is discarded. For the new torsion angles, we calculate the coordinates of atoms N and C^α of residue $d2 + 1$, and sum their distances from the original N and C^α positions. If the resulting distance sum, denoted D_{new}, is smaller than the current sum D, the new torsion angles are accepted. Otherwise, the new angles still have a probability of $e^{(D-D_{new})/T_{t,D}}$ to be accepted, where $T_{t,D} = \frac{T_{0,D}}{1+c_D*t/1000}$, and $T_{0,D} = 1$ and $c_D = 20$ based on test runs. The simulated annealing process stops whenever $D \leq D_{mut}$ (0.3 Å), indicating a smooth connection between the mutated residues and residue $d2$+1.

The crossover and mutation regions are randomly chosen from any section in the length range of L_{xover} and L_{mut} (3-8 for 15 residue macrocycles, 3-10 for 20 residues, and 4-12 for 24 residues). We randomly sort all possible parent pairs, and continue crossover until reaching $s_{thr,xover} = 1.5 * N_{GA}$ children. Similarly, for mutation, we randomly sample 100*N_{GA} backbones from the current population, and continue mutation until reaching $s_{thr,mut} = 1.5 * N_{GA}$ mutants.

ClusterGen stability analyzes were performed on the Greene supercomputer clusters at the New York University's High Performance Computing facilities. In Fig. S5, we plot the computation time spent on different components of ClusterGen, including the initial-population-generating layered simulated annealing, the Matlab part of the genetic algorithm (crossover, mutation, and selection), and the FastRelax part of the genetic algorithm (Cartesian relaxation after crossover and mutation), assuming 96 cores used. The increase in time spent on simulated annealing is due to the expanded initial points. In the genetic algorithm, about 70% of the computation time is spent on Cartesian relaxation.

Algorithm 1 Pseudocode for ClusterGen

Step 1. Generating initial population by simulated annealing

Input: (i) Designed sequence and structure; (ii) Number of simulated annealing initial points for low energy $N_{p,energy}$ and for low RMSD $N_{p,rmsd}$; (iii) Simulated annealing parameters M, $E_{thr,rama}$, $E_{thr,rep}$, $E_{thr,cyc}$, $E_{thr,rmsd}$, $H_{thr,count}$, $E_{cri,rep}$, $E_{cri,cyc}$, $E_{cri,rmsd}$, $H_{cri,count}$, k_0, b, $T_{0,rama}$, $T_{0,rep}$, $T_{0,cyc}$, $T_{0,hbond}$, $T_{0,rmsd}$, c_{rama}, c_{rep}, c_{cyc}, c_{hbond}, c_{rmsd}.

Output: Alternative backbones for the designed sequence.

Randomly select $N_{p,energy}$ initial points from possible combinations of torsion bin centers (Fig. S3) corresponding to the designed sequence ▷ Low energy SA starts
for each initial point **do**
  Perform the layered simulated annealing with Ramachandran energy test, repulsive energy test, cyclic error test, and hydrogen bond energy test as in Algorithm 1
  Record points that satisfy the repulsive energy, cyclic error, and hydrogen bond count criteria as alternative backbones
end for ▷ Low energy SA ends

Randomly select $N_{p,rmsd}$ initial points ▷ Low RMSD SA starts
for each initial point *angles* **do**
  Calculate initial E_{cyc} and backbone-heavy-atom RMSD (E_{rmsd}) from the designed structure
  for time step t from 1 to M **do**
    Generate a random move for each residue within a disk of radius $\frac{k_0}{1+b*t/M}$
    Record the new point *angles_new* generated
    Perform cyclic error test, with parameters $T_{0,cyc}, c_{cyc}, E_{thr,cyc}$
    if cyclic error test passed **then** ▷ RMSD test
      Calculate RMSD $E_{new,rmsd}$ at the new point
      $accept \leftarrow$ False, $T_{t,rmsd} \leftarrow \frac{T_{0,rmsd}}{1+c_{rmsd}*t/M}$
      if $E_{new,rmsd} \leq E_{rmsd}$ **or** $E_{new,rmsd} \leq E_{thr,rmsd}$ **then**
        $accept \leftarrow$ True
      else
        With probability $e^{(E_{rmsd}-E_{new,rmsd})/T_{t,rmsd}}$, set *accept* True
      end if ▷ Metropolis criterion for RMSD ends

      if *accept* is True **then** ▷ Accept the new point
        $angles \leftarrow angles\_new$, $E_{cyc} \leftarrow E_{new,cyc}$, $E_{rmsd} \leftarrow E_{new,rmsd}$
        if $E_{cyc} \leq E_{cri,cyc}$ **and** $E_{rmsd} \leq E_{cri,rmsd}$ **then**
          Record *angles* as an alternative backbone
        end if
      end if ▷ The new point is accepted
    end if ▷ RMSD test ends
  end for
end for ▷ Low RMSD SA ends

Step 2. Torsion angle relaxation (section 5) for alternative backbones

Input: Alternative backbones for the designed sequence.

Output: Relaxed alternative structures with sidechains added and energies computed.

Step 3. Energy-based clustering

Input: (i) Relaxed alternative structures with energies; (ii) Genetic algorithm population N_{GA}; (iii) Clustering RMSD cutoff $rmsd_{cutoff}$ (0.5 Å).

Output: $2 * N_{GA}$ lowest-energy cluster centers.

Sort the N relaxed structures in ascending order of their energies
$library \leftarrow [1, 2, 3, ..., N]$, $centers \leftarrow [\ \ ]$
while $library$ not empty **do**
  $center \leftarrow library(1)$, $members \leftarrow [\ \ ]$
  for each element e in $library$ **do**
    if backbone RMSD between e and $center < rmsd_{cutoff}$ **then**
      add e to $members$
    end if
    remove $members$ from $library$, add $center$ to $centers$
  end for
end while
Record the $2 * N_{GA}$ lowest-energy cluster centers

Step 4. Genetic algorithm

Input: (i) Initial population of $2 * N_{GA}$ lowest-energy cluster centers; (ii) Population size N_{GA}, number of generations M_{GA}, and population decrement R_{GA}; (iii) Crossover parameters: L_{xover}, $s_{thr,xover}$, $rmsd_{xover}$; (iv) Mutation parameters: L_{mut}, $s_{thr,mut}$, D_{mut}, p_{max}, M_{mut}, $T_{0,D}$, C_D.

Output: Alternative structures with energies for the energy landscape.

---

for generation from 1 to M_{GA} **do**
  Crossover children size $s_{xover} \leftarrow 0$ ▷ Crossover starts
  for each pair of two parent backbones in the population **do**
    Randomly select a crossover length from the range L_{xover}
    Randomly select breakpoint residue $b1$, and add length to find breakpoint $b2$

    Obtain the following atoms' coordinates for both parents:
      C^α and C' atoms in residues $b1$ and $b2$
      N and C^α atoms in residues $b1 + 1$ and $b2 + 1$

    Align these atoms from the two parents, and calculate RMSD
    if RMSD $\leq rmsd_{xover}$ **then**
      Exchange residues $b1 + 1$ to $b2$ of the two parents (Fig. S4)
      Record the two crossover children, $s_{xover} \leftarrow s_{xover} + 2$
      Stop the crossover for loop if $s_{xover} \geq s_{thr,xover}$
    end if
  end for ▷ Crossover ends

Randomly sample $100 * N_{GA}$ backbone from the population ▷ Mutation starts
Mutation children size $s_{mut} \leftarrow 0$
for each sample **do**
Randomly select a mutation length from the range L_{mut}
Randomly select the starting residue $d1$, and add length to find the ending residue $d2$

Add random angle perturbations $\leq p_{max}$ to ϕ, ψ of residue $d1$
Calculate new positions of atoms N and C^{α} of residue $d2 + 1$
Sum distances D between these atoms' new positions and their original positions

Step index $t \leftarrow 1$ ▷ Mutation simulated annealing starts
while $D > D_{mut}$ and $t \leq M_{mut}$ **do**
Generate random perturbations $\leq \frac{p_{max}}{1+(p_{max}-1)*t/M_{mut}}$ for ϕ, ψ of residues $d1 + 1$ to $d2$
Set the ϕ, ψ perturbations of a residue to 0 if it leaves the corresponding Ramachandran space
Calculate new positions of atoms N and C^{α} of residue $d2 + 1$
Sum distances D_{new} between these atoms' new positions and their original positions

$T_D \leftarrow \frac{T_{0,D}}{(1+c_D*t/M_{mut})}$ ▷ Metropolis criterion starts
if $D_{new} \leq D$ **then**
Add the random perturbations, $D \leftarrow D_{new}$
else
With probability $e^{(D-D_{new})/T_D}$, add the random perturbations and $D \leftarrow D_{new}$
end if ▷ Metropolis criterion ends

$t \leftarrow t + 1$
end while ▷ Mutation simulated annealing ends

if $D \leq D_{mut}$ **then**
Mutate atom positions in residues $d1$ to $d2$ induced by the angle perturbations
Record the mutant child, $s_{mut} \leftarrow s_{mut} + 1$
Stop the mutation for loop if $s_{mut} \geq s_{thr,mut}$
end if
end for ▷ Mutation ends

Perform FastRelax Cartesian relaxation (**??**) for the crossover and mutant children
Perform energy-based clustering (Step 3)

Select N_{GA} lowest-energy cluster centers as the next generation
Record cluster centers with energies < 0 as alternative structures in the energy landscape
$N_{GA} \leftarrow N_{GA} - R_{GA}$
end for

References

[1] W. Kabsch. A solution for the best rotation to relate two sets of vectors. *Acta Cryst.*, 32:922–923, 1976.
