# Supplementary material for: Heuristic energy-based cyclic peptide design
Source: PLoS Comput Biol. 2025 Apr 30;21(4):e1012290. doi: 10.1371/journal.pcbi.1012290 (PMC12043242; doi:10.1371/journal.pcbi.1012290)
Supplement: S10 Text — (PDF) [file pcbi.1012290.s010.pdf]

10 MD and REMD protocol

Our molecular dynamics (MD) simulation protocol started with a rapid energy minimization, which used the Particle Mesh Ewald method[1] for long-range electrostatic interactions with a cutoff of 1 nm, and constrained the lengths of all bonds that involved a hydrogen atom. The water molecules were rigid. A leapfrog Verlet integrator was used with timestep of 1 fs.

After energy minimization, we conducted a 100 ps constant volume and temperature (NVT) ensemble equilibration using Langevin middle integrator, which does the LFMiddle discretization.[2] The temperature was set to be 300 K, the collision rate 1/ps, and the timestep 2 fs. Finally, we ran a 1 μs constant pressure and temperature (NPT) ensemble using the same Langevin middle integrator setting, together with a Monte Carlo Barostat[3,4] at pressure 1 bar. We wrote trajectory frames every 10 ps. For trajectory analysis and RMSD calculations, wed use the Python package *MDAnalysis*.[5]

For replica exchange molecular dynamics (REMD) simulations, the initial preparation involved solvation, energy minimization, and equilibration within an NVT ensemble, following the same protocols as described previously. The prepared system was then replicated across a series of temperatures ranging from 300 K to 500 K. We chose the temperatures using a webserver[6] to have a predicted exchange probability of 0.25.

A constant pressure of 1 bar was maintained for all replicas. During each iteration, 1000 steps of Langevin dynamics integration were performed with collision rate 1/ps and timestep 2 fs. At the end of the iteration, replicas with neighboring temperatures had a chance to swap, following the Metropolis criterion.[7] We ran an initial 50000 iterations (100 ns) and checked convergence using the all-atom peptide radius of gyration (Rg) computed by the Python package *MDTraj v1.9.8*.[8]

To assess the REMD convergence, we adopted the temperature and radius of gyration analyses outlined in.[9] For temperature, we checked whether the replicas have sufficiently explored the various temperature states. Fig. S12 displays temperature trajectories of the first and last replicas in each simulation. Fig. S13 shows the percentage of time each replica spends in different temperature states. The data indicate that the replicas comprehensively traverse the entire temperature ranges, spending relatively even time across the states.

For radius of gyration (Rg), we plot Rg distributions from two distinct time intervals in Fig. S14. Simulations of PDB 6uf7, LowEnergy 19384, and the random sequences of 6uf7, 169032, and 31759, achieve observable Rg overlaps before 100 ns. For other simulations, the extension of runtime leads to Rg overlaps later on, with exceptions of Design 136805, Design 3114, LowEnergy 83218, LowEnergy 19384, LowEnergy 31759, and random sequence 31759. For these six cases, we analyze their average RMSDs over time and observe convergence, as illustrated in Fig. S15. Consequently, we conclude our REMD simulations as converged.

References

[1] U. Essmann, L. Perera, M. Berkowitz, T. Darden, H. Lee, and L. Pedersen. A smooth particle mesh Ewald method. *J. Chem. Phys.*, 103:8577–8593, 1995.

[2] Z. Zhang, X. Liu, K. Yan, M. Tuckerman, and J. Liu. Unified efficient thermostat scheme for the canonical ensemble with holonomic or isokinetic constraints via molecular dynamics. *J. Phys. Chem. A*, 123:6056–6079, 2019.

[3] K. Chow and D. Ferguson. Isothermal-isobaric molecular dynamics simulations with Monte Carlo volume sampling. *Comput. Phys. Commun.*, 91:283–289, 1995.

[4] J. Åqvist, P. Wennerström, M. Nervall, S. Bjelic, and B. Brandsdal. Molecular dynamics simulations of water and biomolecules with a Monte Carlo constant pressure algorithm. *Chem. Phys. Lett.*, 384:288–294, 2004.

[5] N. Michaud-Agrawal, E. Denning, T. Woolf, and O. Beckstein. Mdanalysis: A toolkit for the analysis of molecular dynamics simulations. *J. Comput. Chem.*, 32:2319–2327, 2011.

[6] A. Patriksson and D. Spoel. A temperature predictor for parallel tempering simulations. *Phys. Chem. Chem. Phys.*, 10:2073–2077, 2008.

[7] Y. Sugita and Y. Okamoto. Replica-exchange molecular dynamics method for protein folding. *Chem. Phys. Lett.*, 314:141–151, 1999.

[8] R. McGibbon, K. Beauchamp, M. Harrigan, C. Klein, J. Swails, et al. Mdtraj: A modern open library for the analysis of molecular dynamics trajectories. *Biophys. J.*, 109:1528–1532, 2015.

[9] R. Qi, G. Wei, B. Ma, and R. Nussinov. Replica exchange molecular dynamics: A practical application protocol with solutions to common problems and a peptide aggregation and self-assembly example. *Methods Mol Biol*, 1777:101–119, 2018.
