# Supplementary material for: Heuristic energy-based cyclic peptide design
Source: PLoS Comput Biol. 2025 Apr 30;21(4):e1012290. doi: 10.1371/journal.pcbi.1012290 (PMC12043242; doi:10.1371/journal.pcbi.1012290)

Figure S1: **Backbone energy functions.** (A) Example repulsive, attractive, electrostatic, and isotropic solvation energies between backbone atom N and atom C′ are plotted against their distance. (B) The three components of hydrogen bond energy. All energies are in units of kcal/mol.

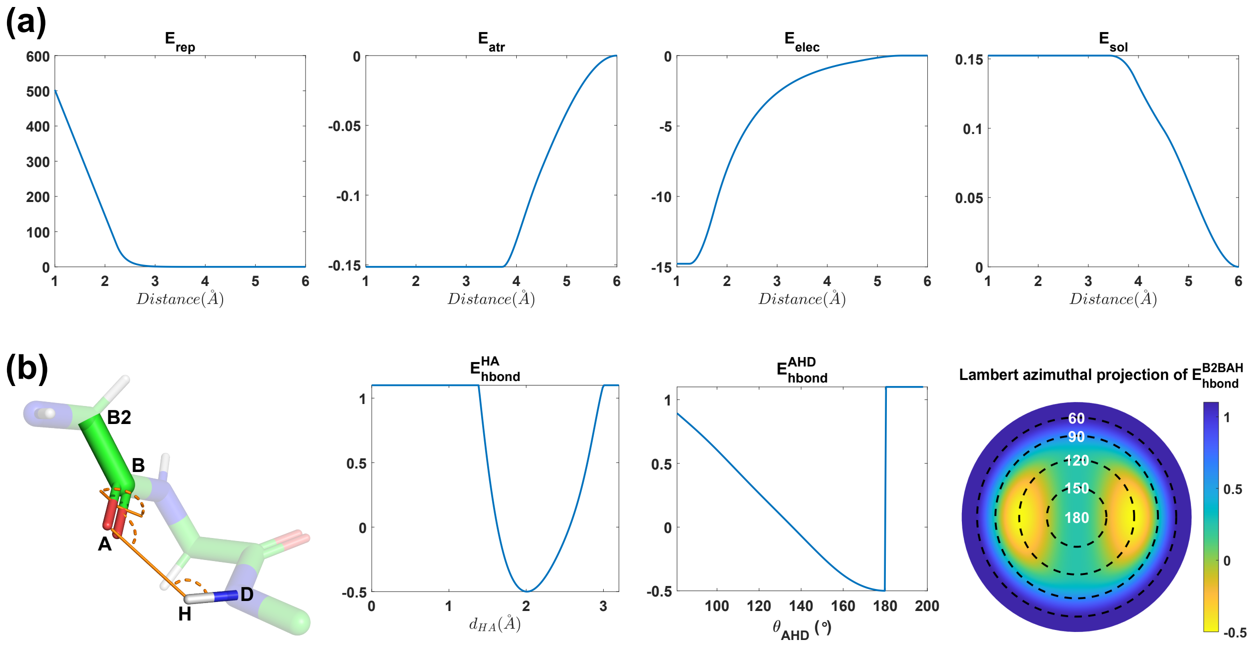

Supplement: S1 Fig — (PDF) [file pcbi.1012290.s011.pdf]
