# Supplementary material for: Heuristic energy-based cyclic peptide design
Source: PLoS Comput Biol. 2025 Apr 30;21(4):e1012290. doi: 10.1371/journal.pcbi.1012290 (PMC12043242; doi:10.1371/journal.pcbi.1012290)

Figure S2: **Extended glycine Ramachandran space sampling.** (A) The Ramachandran space is partitioned into six torsion bins, with centers marked and Ramachandran energy (kcal/mol) plotted. A partial simulated annealing path of a residue is drawn for illustration, starting from center 2 with a random move disk of radius k_0. At time step t, the random move disk shrinks to a radius of k_t. (B) An example initial 7-residue polyglycine chain has all torsion angles chosen as center 1. An example good configuration after simulated annealing is labeled with hydrogen bonds and energies. All energies are in units of kcal/mol.

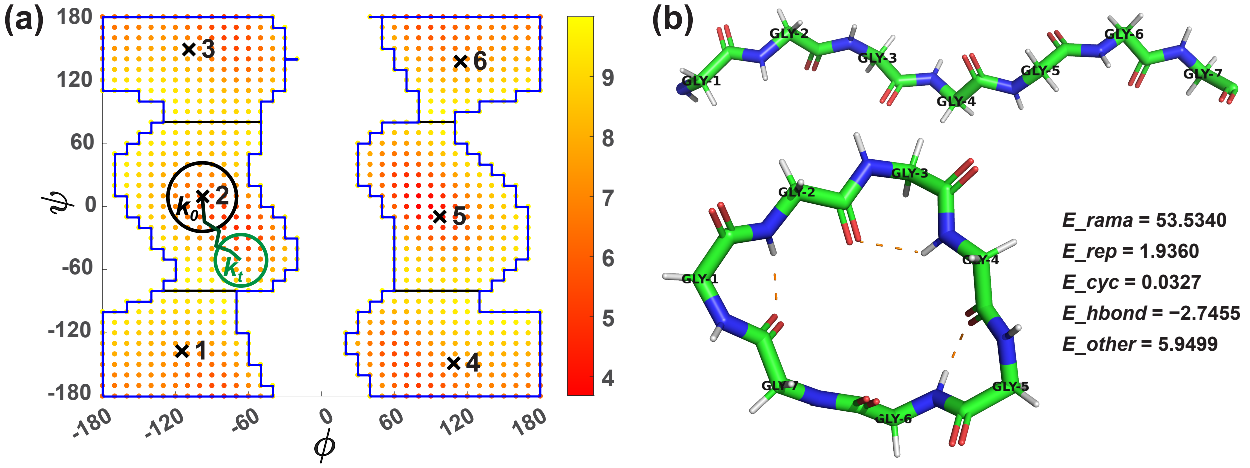

Supplement: S2 Fig — (PDF) [file pcbi.1012290.s012.pdf]
