# Supplementary material for: Heuristic energy-based cyclic peptide design
Source: PLoS Comput Biol. 2025 Apr 30;21(4):e1012290. doi: 10.1371/journal.pcbi.1012290 (PMC12043242; doi:10.1371/journal.pcbi.1012290)

Figure S3: **Ramachandran spaces for L and D amino acids.** The six different dypes of Ramachandran spaces correspond to L- and D-proline, L- and D-valine (also isoleucine and threonine), and L- and D-alanine (also remaining amino acids). The Ramachandran space centers are marked and the Ramachandran energies are in units of kcal/mol.

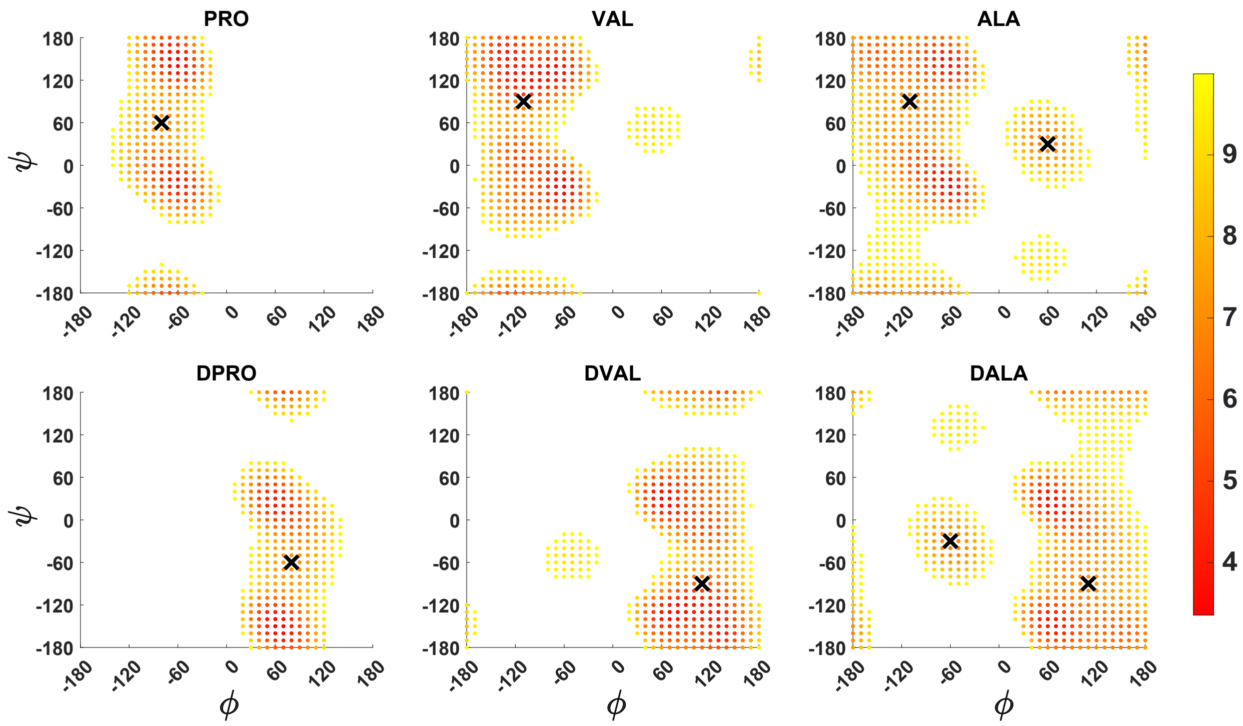

Supplement: S3 Fig — (PDF) [file pcbi.1012290.s013.pdf]
