# Supplementary material for: Heuristic energy-based cyclic peptide design
Source: PLoS Comput Biol. 2025 Apr 30;21(4):e1012290. doi: 10.1371/journal.pcbi.1012290 (PMC12043242; doi:10.1371/journal.pcbi.1012290)

Figure S4: **ClusterGen crossover and mutation.** (A) Example crossover of two 15-residue backbones. (B) Example mutation of a 15-residue backbone.

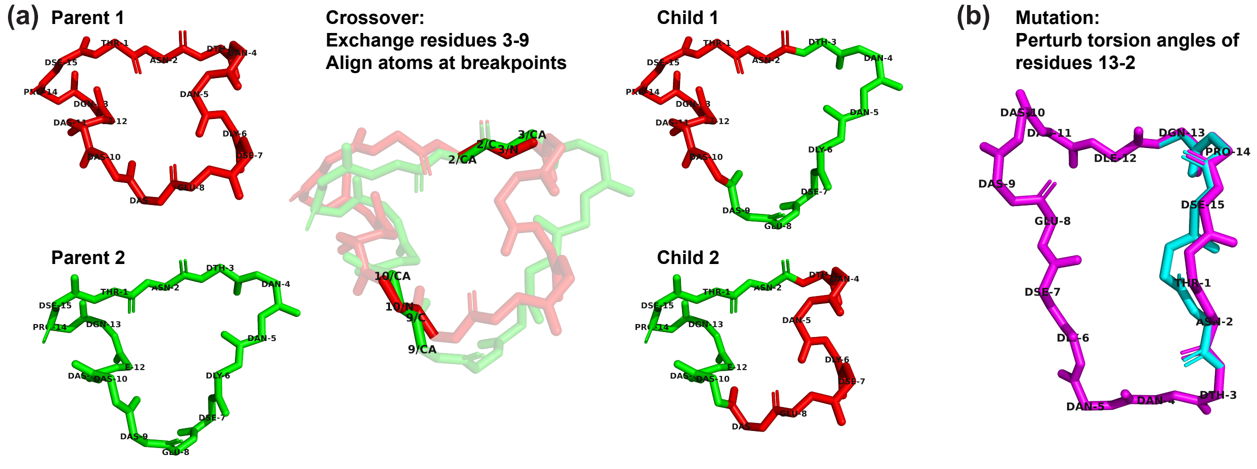

Supplement: S4 Fig — (PDF) [file pcbi.1012290.s014.pdf]
