# Supplementary material for: Heuristic energy-based cyclic peptide design
Source: PLoS Comput Biol. 2025 Apr 30;21(4):e1012290. doi: 10.1371/journal.pcbi.1012290 (PMC12043242; doi:10.1371/journal.pcbi.1012290)

Figure S5: **ClusterGen computation time breakdown.** For macrocycle designs of 15, 20, and 24 residues, we breakdown the computation CPU hours per ClusterGen stability analysis, using NYU Greene HPC.

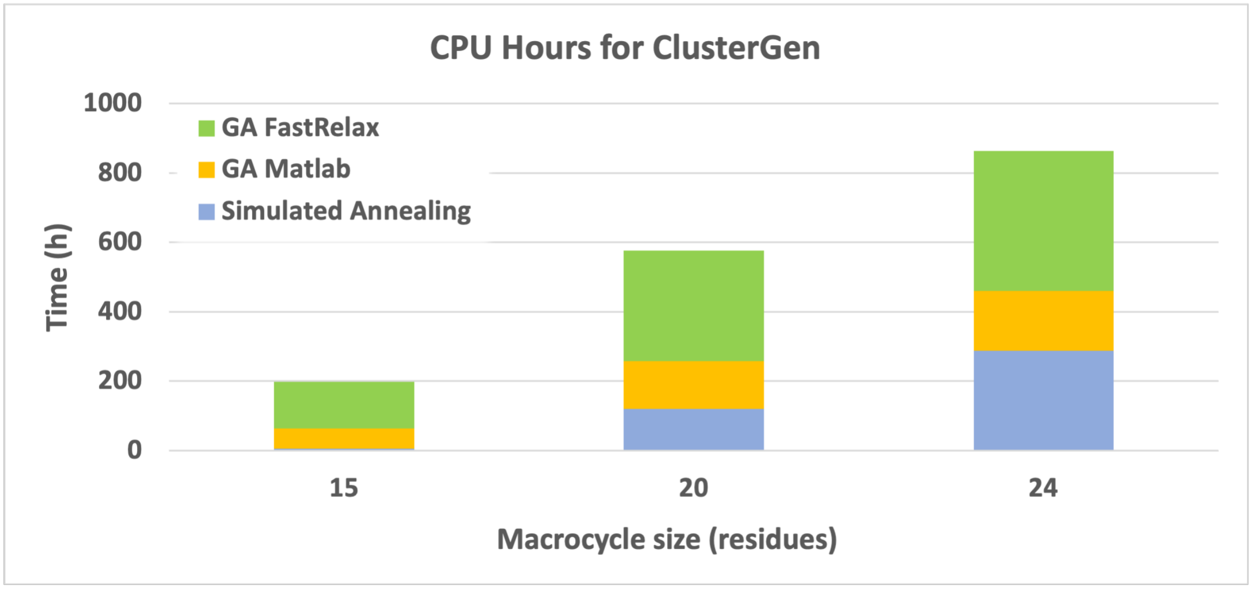

Supplement: S5 Fig — (PDF) [file pcbi.1012290.s015.pdf]
