# Supplementary material for: Heuristic energy-based cyclic peptide design
Source: PLoS Comput Biol. 2025 Apr 30;21(4):e1012290. doi: 10.1371/journal.pcbi.1012290 (PMC12043242; doi:10.1371/journal.pcbi.1012290)
Supplement: S6 Fig — (PDF) [file pcbi.1012290.s016.pdf]

Figure S6: **CyclicChamp design results.** We list the number of structures obtained in each step of our CyclicChamp design workflow. For relaxed backbones and designed sequences, we show the number of structures with energies below the threshold (in kcal/mol). For high P_{Near} candidates, we show the number of designs having $P_{Near} > 0.9$ out of the total validated designs, with the highest P_{Near} score written below.

n	Initial Points	Backbones	Clusters	Low-Energy Clusters	Designed Sequences	High Pnear (>0.9) Candidates			
						simple_cycpep_predict	Rama-stability filtering	ClusterGen	Reshape
7	39,996	5,277,863	12,196	6,981 (<8)	513 (<−8)	38/513 (0.981)	77/513 (0.986)	–	–
15	100,000	640,477	192,864	74,626 (<0)	1,103 (<−30)	3/75 (0.967)	–	9/75 (0.958)	–
20	100,000	983,567	157,217	81,718 (<0)	97 (<−40)	0/22 (0.172)	–	0/69 (0.897)	22/69 (0.982)
24	100,000	186,483	42,758	26,537 (<0)	105 (<−45)	0/11 (0.042)	–	0/82 (0.896)	14/82 (0.991)
