# Supplementary material for: Heuristic energy-based cyclic peptide design
Source: PLoS Comput Biol. 2025 Apr 30;21(4):e1012290. doi: 10.1371/journal.pcbi.1012290 (PMC12043242; doi:10.1371/journal.pcbi.1012290)

Figure S7: **D-amino acid counts in CyclicChamp designs.** For each macrocycle size, we show the distribution of designs that have various number of D-amino acids. All designs have mixed chirality.

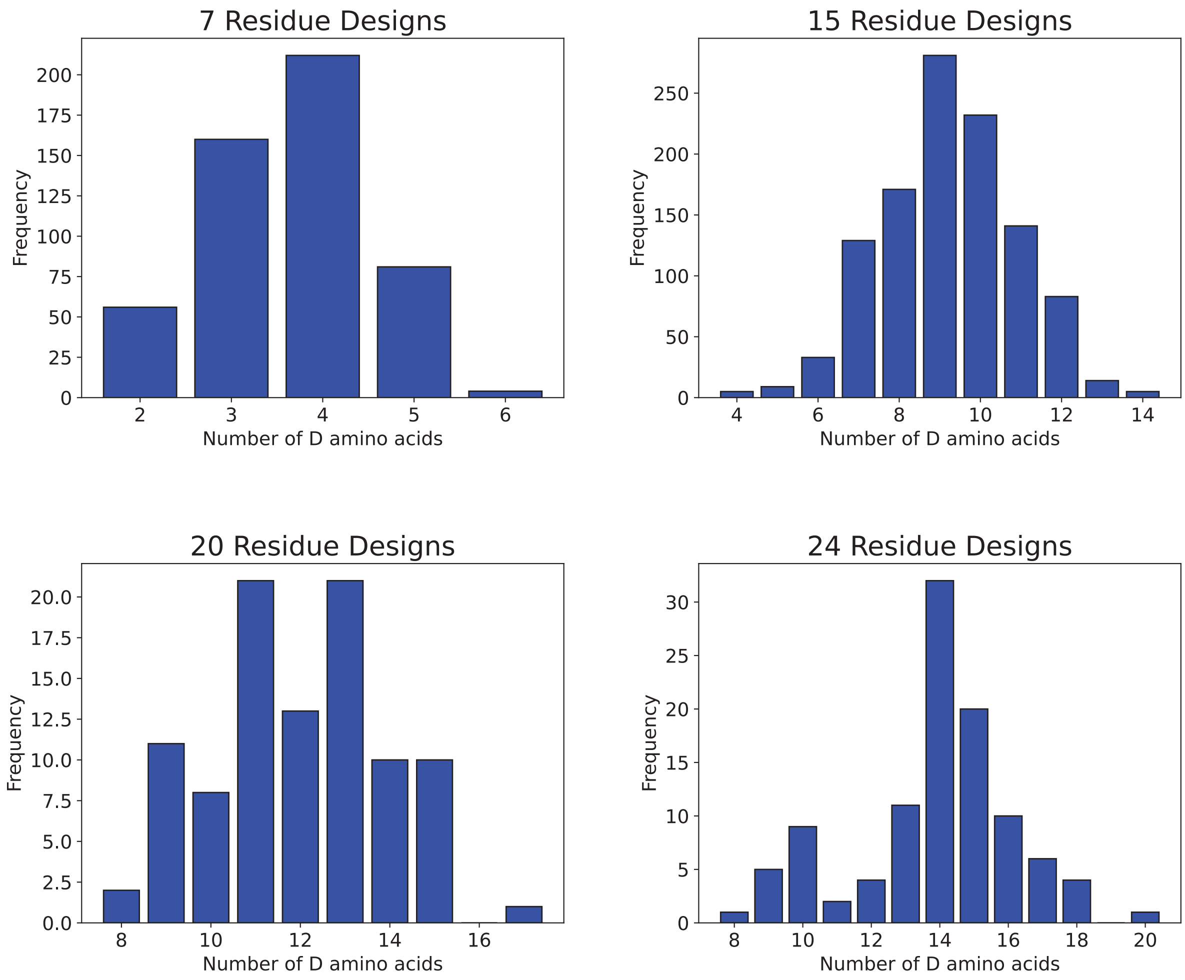

Supplement: S7 Fig — (PDF) [file pcbi.1012290.s017.pdf]
