# Supplementary material for: Heuristic energy-based cyclic peptide design
Source: PLoS Comput Biol. 2025 Apr 30;21(4):e1012290. doi: 10.1371/journal.pcbi.1012290 (PMC12043242; doi:10.1371/journal.pcbi.1012290)

Figure S8: **Top 20-residue designs shown in sphere mode.** Prolines are colored in purple, and hydrophobic amino acids (ALA, ILE, LEU, VAL, MET, PHE) colored in orange.

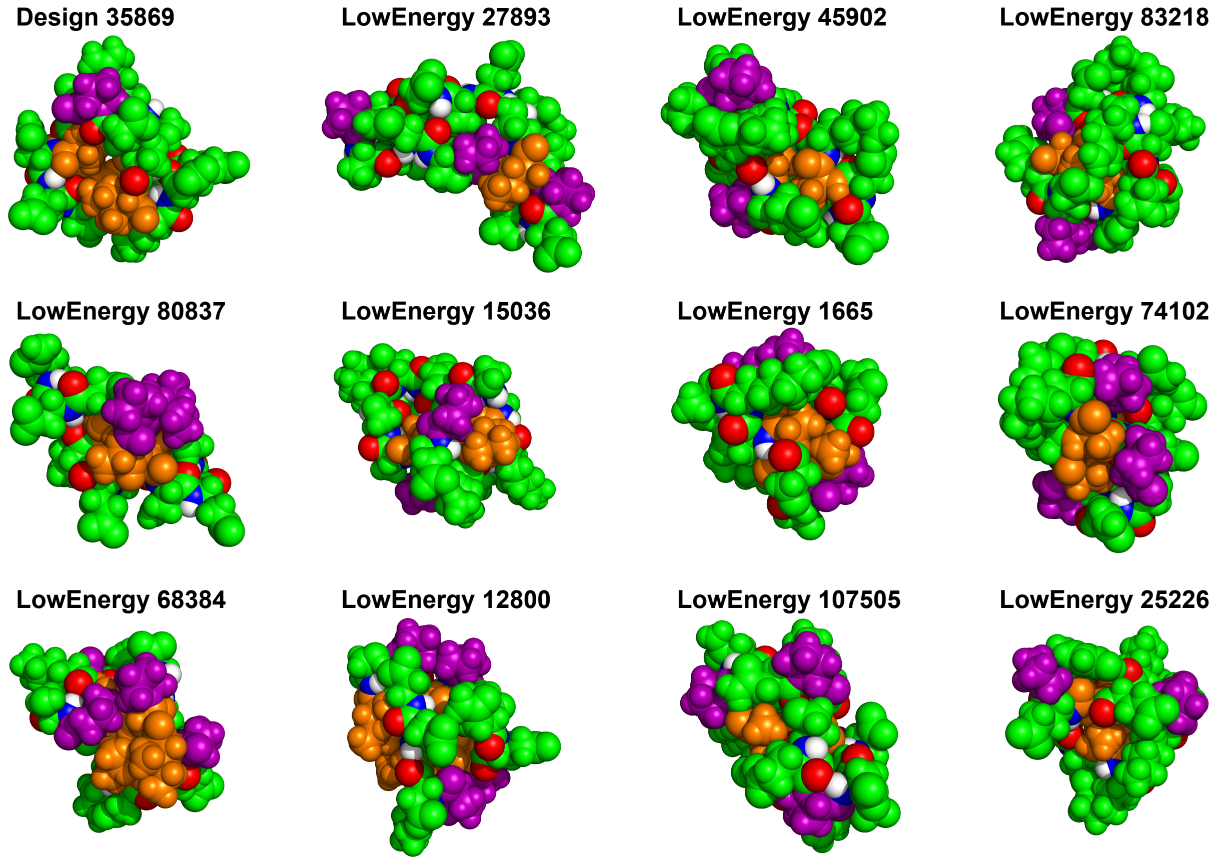

Supplement: S8 Fig — (PDF) [file pcbi.1012290.s018.pdf]
