# Supplementary material for: Heuristic energy-based cyclic peptide design
Source: PLoS Comput Biol. 2025 Apr 30;21(4):e1012290. doi: 10.1371/journal.pcbi.1012290 (PMC12043242; doi:10.1371/journal.pcbi.1012290)

Figure S10: **Molecular dynamics simulation stable trajectories.** RMSDs are calculated between the backbone C^{α} atoms of the trajectory frames and our designed structures.

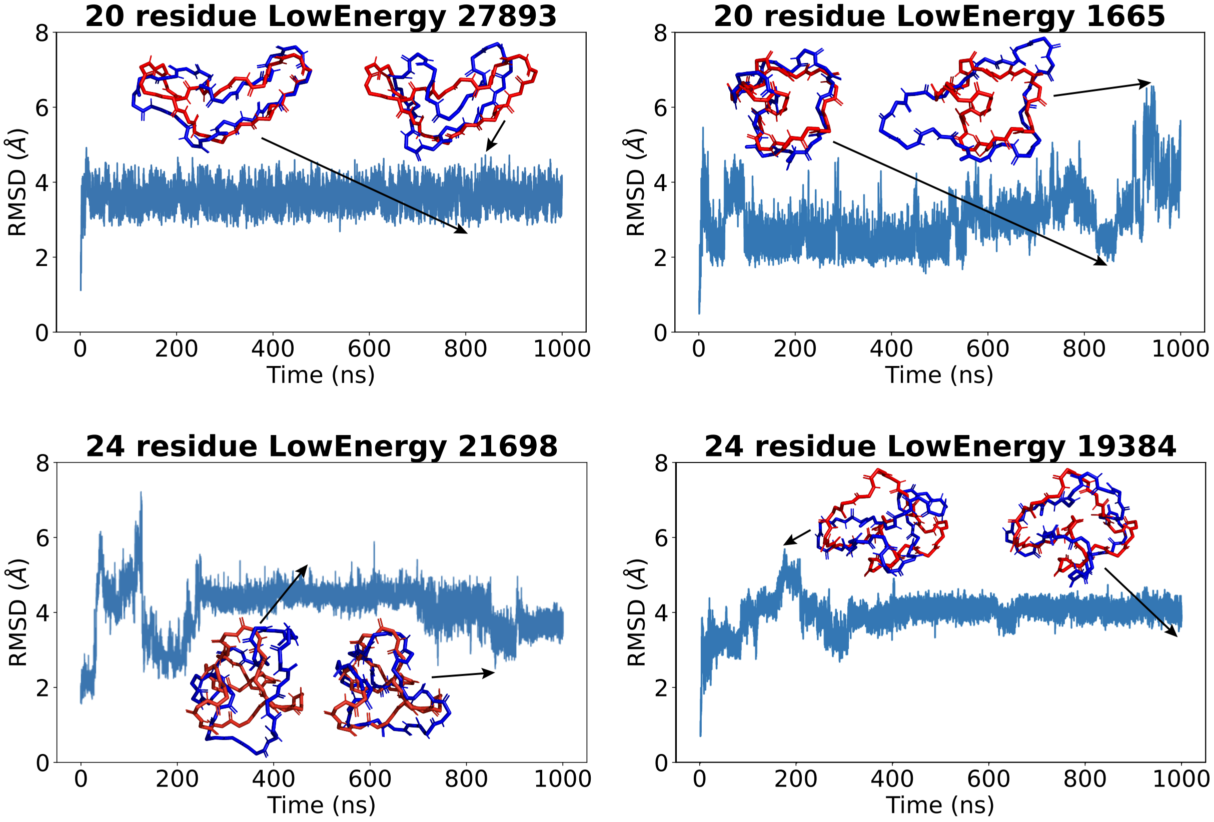

Supplement: S10 Fig — (PDF) [file pcbi.1012290.s020.pdf]
