# Supplementary material for: Heuristic energy-based cyclic peptide design
Source: PLoS Comput Biol. 2025 Apr 30;21(4):e1012290. doi: 10.1371/journal.pcbi.1012290 (PMC12043242; doi:10.1371/journal.pcbi.1012290)

Figure S13: **REMD temperature dwell time plots.** The percentages of simulation time spent on each temperature are drawn for all replicas, with the standard deviations shown as error bars.

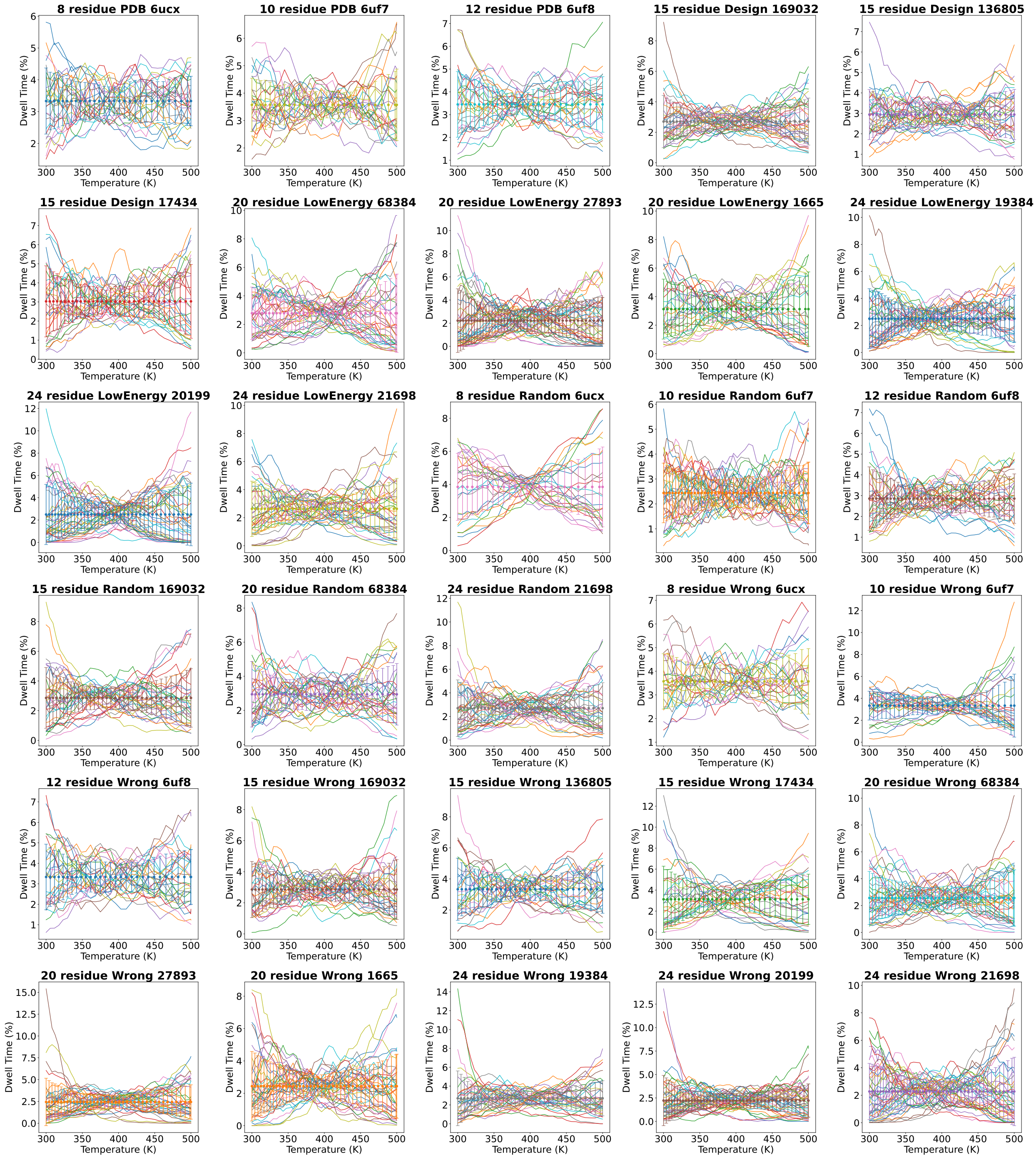

Supplement: S13 Fig — (PDF) [file pcbi.1012290.s023.pdf]
