# Supplementary material for: Heuristic energy-based cyclic peptide design
Source: PLoS Comput Biol. 2025 Apr 30;21(4):e1012290. doi: 10.1371/journal.pcbi.1012290 (PMC12043242; doi:10.1371/journal.pcbi.1012290)

Figure S14: **REMD convergence check of Rg.** Distributions of all-atom peptide radii of gyration are plotted for two different time intervals.

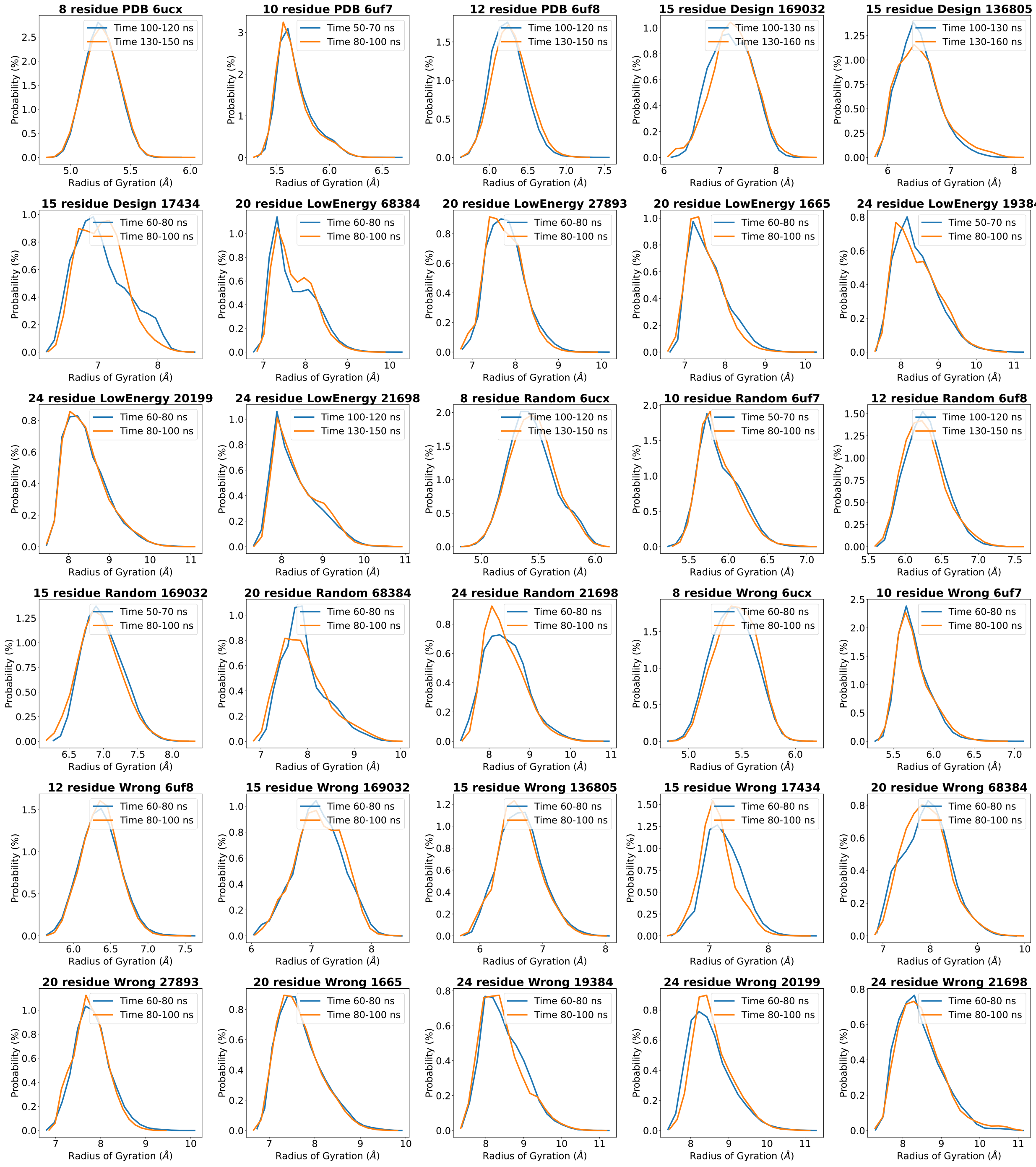

Supplement: S14 Fig — (PDF) [file pcbi.1012290.s024.pdf]
