# Supplementary material for: Heuristic energy-based cyclic peptide design
Source: PLoS Comput Biol. 2025 Apr 30;21(4):e1012290. doi: 10.1371/journal.pcbi.1012290 (PMC12043242; doi:10.1371/journal.pcbi.1012290)

Figure S15: **REMD convergence check of RMSD.** For simulations that do not show a good overlap of Rg distributions from the two time intervals, we plot the average C^{α}-atom RMSDs between trajectory frames sampled at temperature state 300 K and our designed structure over simulation time.

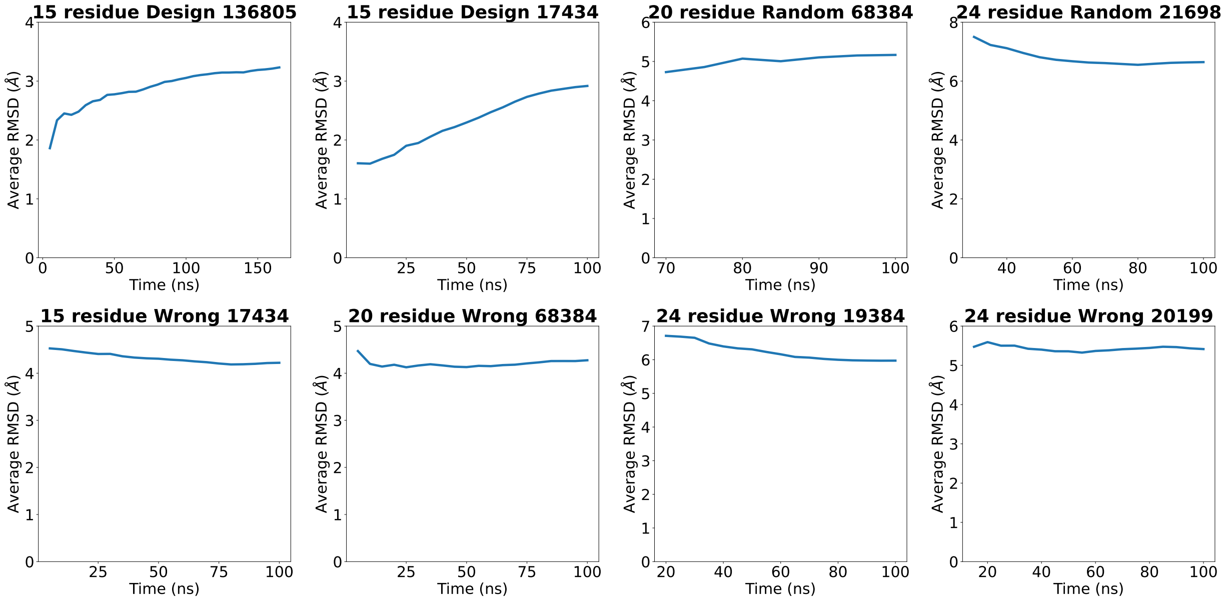

Supplement: S15 Fig — (PDF) [file pcbi.1012290.s025.pdf]
