# Supplementary material for: Heuristic energy-based cyclic peptide design
Source: PLoS Comput Biol. 2025 Apr 30;21(4):e1012290. doi: 10.1371/journal.pcbi.1012290 (PMC12043242; doi:10.1371/journal.pcbi.1012290)

Figure S16: **REMD free energy surfaces with negative controls.** (a) As a negative control, for each size, we selected one design and randomly permuteed its sequence. The FES comparisons are shown. (b) For our 15-24 residue designs, we re-ran the REMD simulation from a distinct starting structure (colored in green) chosen from the P_{Near} landscape, which has low energy and high RMSD from the design (2.1-6.8 Å).

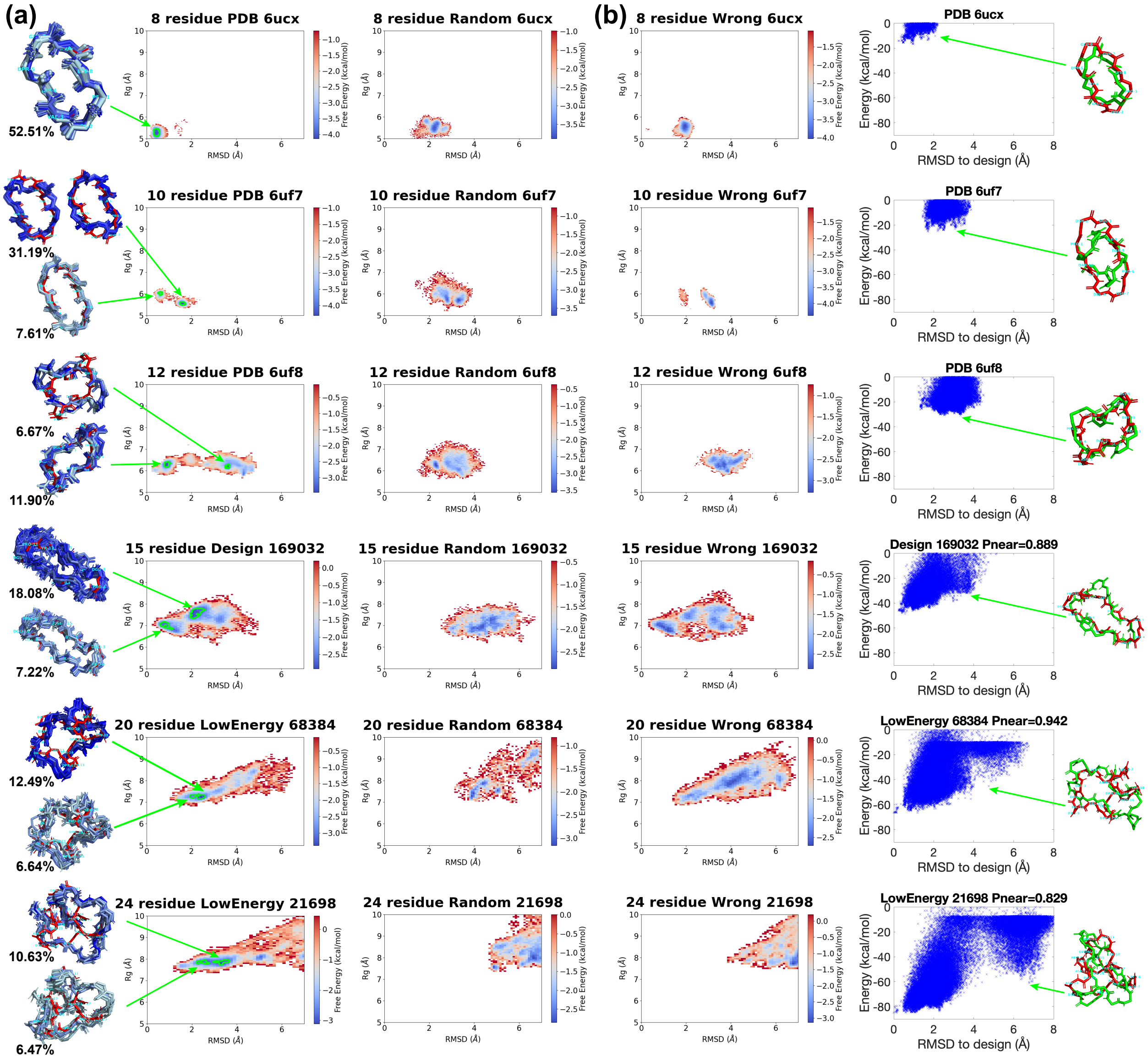

Supplement: S16 Fig — (PDF) [file pcbi.1012290.s026.pdf]
