# Supplementary material for: Heuristic energy-based cyclic peptide design
Source: PLoS Comput Biol. 2025 Apr 30;21(4):e1012290. doi: 10.1371/journal.pcbi.1012290 (PMC12043242; doi:10.1371/journal.pcbi.1012290)

Figure S17: **Other REMD free energy surfaces.** (a) Representative structures are shown for the energy basins. (b) REMD simulations re-run from distinct starting structures (colored in green) chosen from the P_{Near} landscapes, which have low energies and high RMSDs from the designs (3.8-6.8 Å).

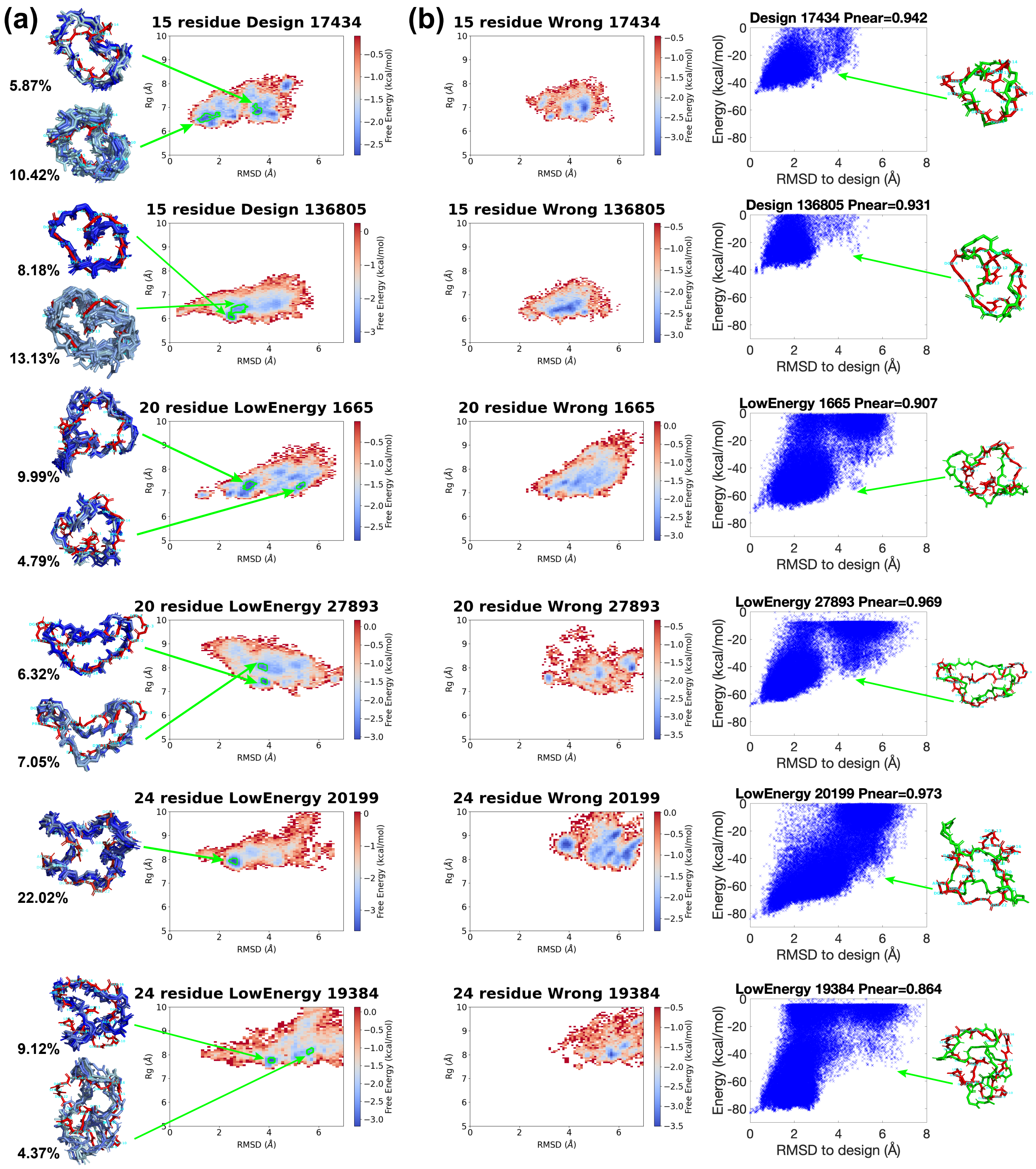

Supplement: S17 Fig — (PDF) [file pcbi.1012290.s027.pdf]
