# Supplementary material for: Heuristic energy-based cyclic peptide design
Source: PLoS Comput Biol. 2025 Apr 30;21(4):e1012290. doi: 10.1371/journal.pcbi.1012290 (PMC12043242; doi:10.1371/journal.pcbi.1012290)

Figure S18: **Energy landscape predictions of available macrocycle structures deposited in the PDB.** In each landscape, the predicted low-energy cluster centers are marked in red. Rosetta energy scores of the PDB structures are drawn as dash lines for reference.

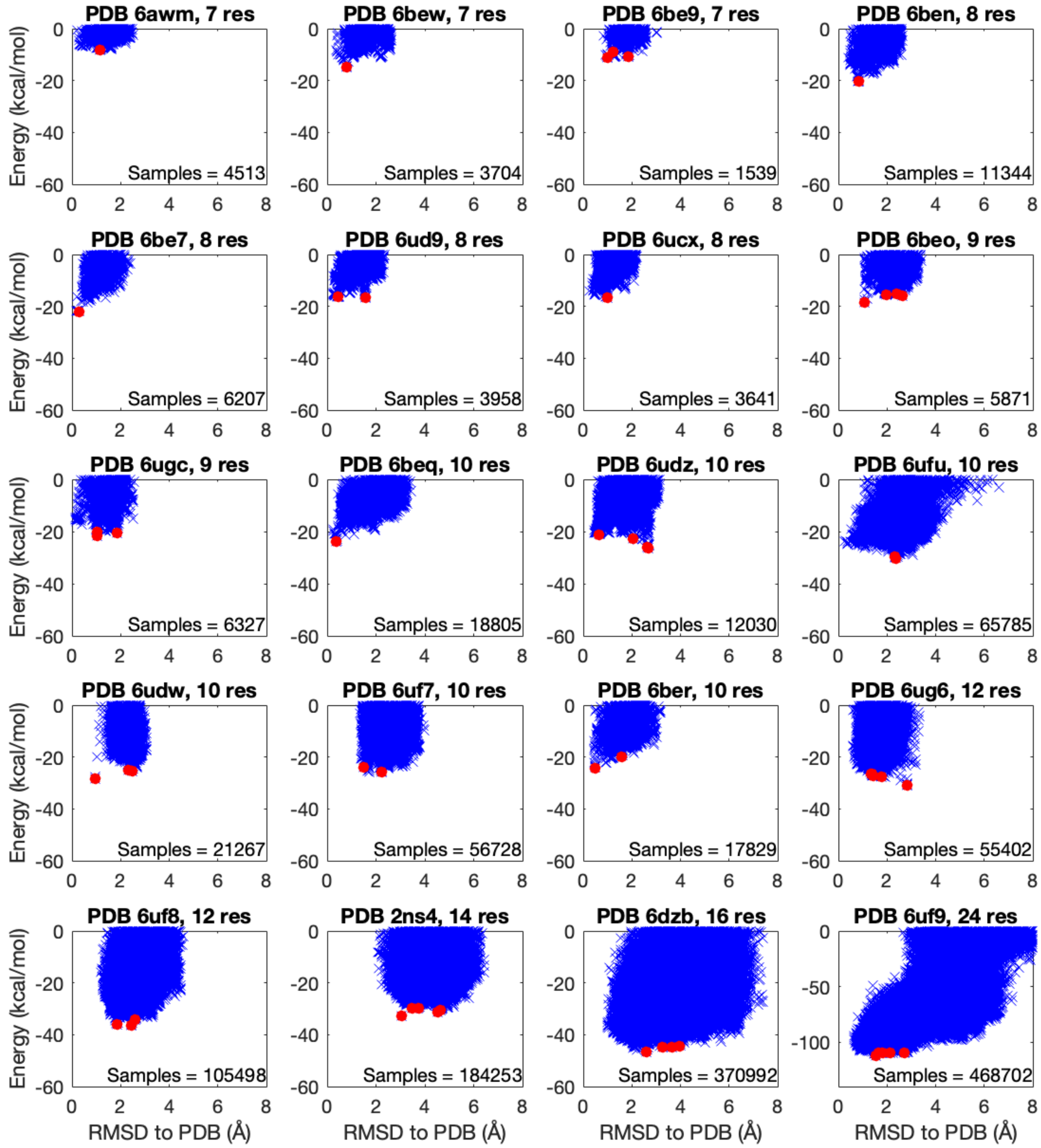

Supplement: S18 Fig — (PDF) [file pcbi.1012290.s028.pdf]
