# Supplementary material for: Heuristic energy-based cyclic peptide design
Source: PLoS Comput Biol. 2025 Apr 30;21(4):e1012290. doi: 10.1371/journal.pcbi.1012290 (PMC12043242; doi:10.1371/journal.pcbi.1012290)
Supplement: S19 Fig — (PDF) [file pcbi.1012290.s029.pdf]

Figure S19: **Other structure predictions for available macrocycle structures deposited in the PDB.** Predicted low-energy cluster centers (green) are aligned to the PDB structures (orange), with RMSDs shown. PDB structures from the 2017[1] and 2020[2] Rosetta paper are labeled in blue and black, respectively, and all other structures in pink.

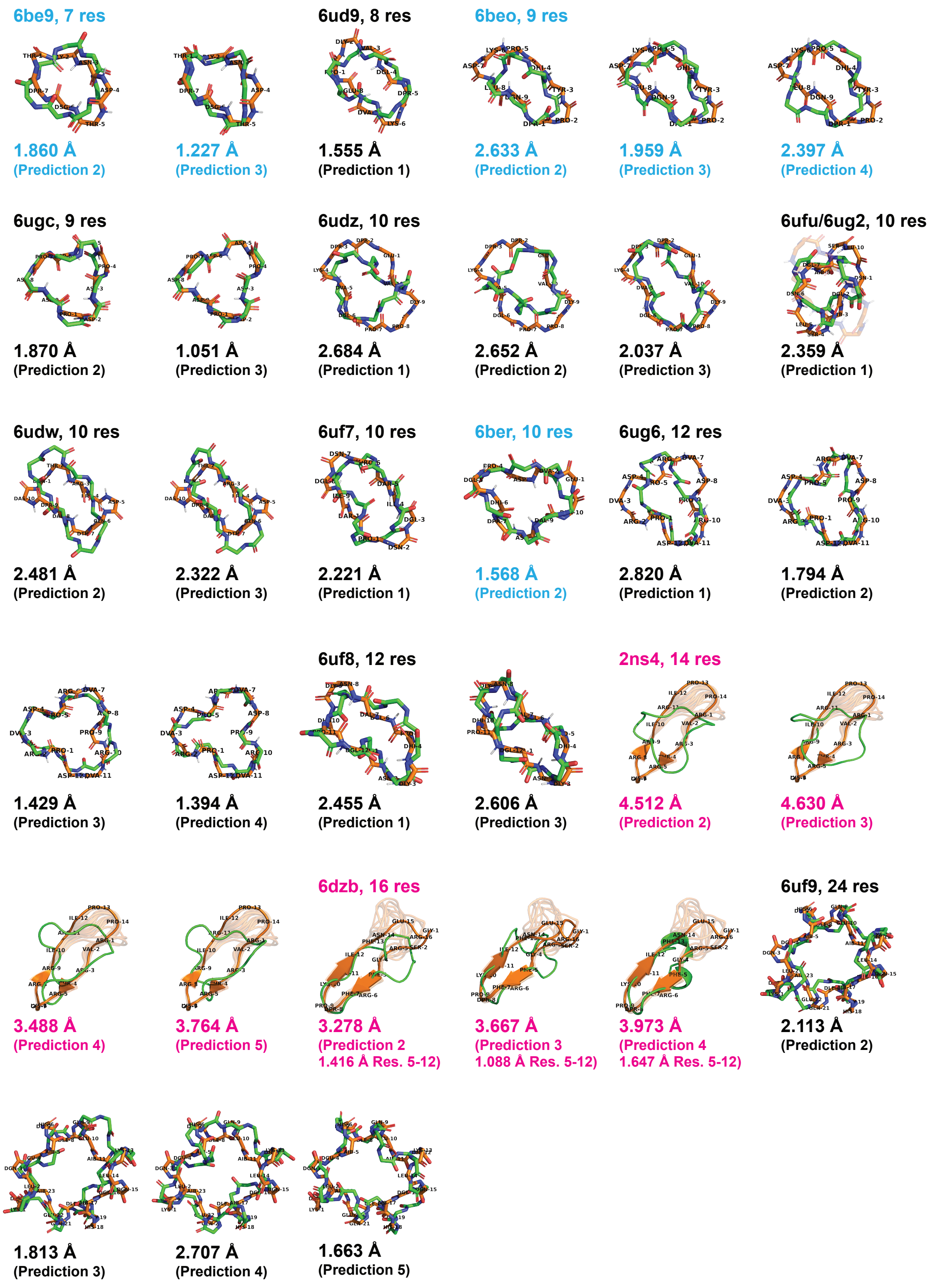

References

[1] P. Hosseinzadeh, G. Bhardwaj, V. Mulligan, M. Shortridge, T. Craven, F. Pardo-Avila, S. Rettie, et al. Comprehensive computational design of ordered peptide macrocycles. *Science*, 358:1461–1466, 2017.

[2] V. Mulligan, C. Kang, M. Sawaya, S. Rettie, X. Li, I. Antselovich, T. Craven, A. Watkins, J. Labonte, F. DiMaio, T. Yeates, and D. Baker. Computational design of mixed chirality peptide macrocycles with internal symmetry. *Protein Sci*, 29:2433–2445, 2020.
