# Supplementary material for: Heuristic energy-based cyclic peptide design
Source: PLoS Comput Biol. 2025 Apr 30;21(4):e1012290. doi: 10.1371/journal.pcbi.1012290 (PMC12043242; doi:10.1371/journal.pcbi.1012290)
Supplement: S1 Table — (PDF) [file pcbi.1012290.s030.pdf]

Table S1: **Backbone simulated annealing parameters.** For the four peptide sizes, the thresholds for passing energy tests, the criteria for good backbone candidates, the disk radius parameters for random moves, and the initial temperatures and temperature dropping rates are given.

| ***n*** | **Energy thresholds** | | | | | **Candidate criteria** | | |
|---|---|---|---|---|---|---|---|---|
| | $E_{thr,rama}$ | $E_{thr,rep}$ | $E_{thr,cyc}$ | $H_{thr,count}$ | $E_{thr,other}$ | $E_{cri,rep}$ | $E_{cri,cyc}$ | $H_{cri,count}$ |
| **7** | 56 | 10 | 0.3 | ≥3 | 10 | 5 | 0.3 | ≥3 |
| **15** | 120 | 15 | 0.3 | ≥5 | – | 10 | 0.3 | ≥5 |
| **20** | 160 | 18 | 1 | ≥7 | – | 13 | 1 | ≥7 |
| **24** | 192 | 20 | 1 | ≥8 | – | 15 | 1 | ≥8 |

| **Radius** | | **Initial temperatures** | | | | | **Dropping rates** | | | | |
|---|---|---|---|---|---|---|---|---|---|---|---|
| $k_0$ | $b$ | $T_{0,rama}$ | $T_{0,rep}$ | $T_{0,cyc}$ | $T_{0,hbond}$ | $T_{0,other}$ | $c_{rama}$ | $c_{rep}$ | $c_{cyc}$ | $c_{hbond}$ | $c_{other}$ |
| 0.9 | 15 | 10 | 20 | 2 | 2 | 20 | 4 | 14 | 16 | 20 | 6 |
| 0.7 | 16 | 20 | 60 | 4 | 4 | – | 2 | 14 | 16 | 18 | – |
| 0.6 | 18 | 25 | 80 | 5 | 5 | – | 4 | 16 | 18 | 22 | – |
| 0.6 | 18 | 30 | 100 | 6 | 6 | – | 4 | 14 | 20 | 22 | – |
