# Supplementary material for: Heuristic energy-based cyclic peptide design
Source: PLoS Comput Biol. 2025 Apr 30;21(4):e1012290. doi: 10.1371/journal.pcbi.1012290 (PMC12043242; doi:10.1371/journal.pcbi.1012290)
Supplement: S2 Table — (PDF) [file pcbi.1012290.s031.pdf]

Table S2: Amino acid sequences of designs having high P_{Near} values.

Top 7 Residue Designs	
**Name**	**Sequence**
Design5191	PRO-DPRO-PRO-DLYS-GLU-DASP-DHIS
Design10566	VAL-DASN-GLU-DGLU-DPRO-PRO-DLYS
Design5790	LYS-THR-DILE-DPRO-PRO-DVAL-ASP
Design9104	PRO-DPRO-GLU-DGLN-GLU-ASN-DLYS
Design2787	ASP-LYS-THR-DILE-DPRO-PRO-DVAL
Design2198	ASN-DLYS-SER-GLU-DPRO-DTYR-DPRO
Design10632	PRO-DPRO-LYS-DGLN-ASP-GLU-DGLN
Design372	ASP-GLU-DPRO-DTYR-DPRO-ASN-DLYS
Design5982	PRO-PRO-DPRO-GLU-DALA-LYS-DASN
Design9541	DASN-PRO-DPRO-GLU-DLYS-LEU-DGLU
Design11103	DASN-DPRO-ASN-DLYS-TYR-DPRO-GLU
Design1058	GLU-DHIS-PRO-DPRO-DASP-DASP-DLYS
Design3860	DSER-DLYS-PRO-DASN-DPRO-GLU-DVAL
Design8906	PRO-ARG-DPRO-DARG-ASN-DASN-ASP
Design8145	VAL-DASN-DLYS-DTHR-GLU-PRO-DPRO
Design2547	VAL-DASN-DLYS-DSER-GLU-PRO-DPRO
Design5063	GLU-DVAL-DPRO-DASN-PRO-DLYS-DPRO
Design9191	PRO-ASN-DASN-GLU-DPRO-DLYS-SER
Design7437	VAL-ASN-DASN-GLU-GLU-DPRO-DLYS
Design4816	ASN-DSER-GLU-DPRO-LYS-PRO-THR
Design878	DSER-GLU-DPRO-LYS-PRO-THR-ASN
Design9772	GLU-DPRO-PRO-ARG-ASN-DTHR-SER
Design9974	DGLU-PRO-LYS-DVAL-DASN-GLU-ASN
Design5863	PRO-GLU-DSER-DPRO-DASN-ASN-DLYS
Design1828	SER-PRO-ASN-DASN-GLU-DPRO-DLYS
Design1644	DGLN-DPRO-PRO-THR-DASN-GLU-DARG
Design3836	PRO-GLU-DASN-LYS-DASN-THR-GLU
Design3227	PRO-THR-DASN-GLU-DARG-DGLN-DPRO
Design5621	PRO-ASP-LYS-DASN-VAL-ALA-DPRO
Design7997	GLU-PRO-GLU-DASN-LYS-DASN-THR
Design151	PRO-ASN-GLU-DLYS-VAL-ALA-DPRO
Design12024	DASN-ASN-DLYS-PRO-GLU-DSER-DPRO

Top 15 Residue Designs	
**Name**	**Sequence**
Design169032	DTYR-DILE-DGLU-PRO-DVAL-ILE-PRO-SER-DSER-DGLU-PRO-TYR-DLYS-GLU-SER
Design3114	ASP-GLU-SER-SER-DASN-DALA-DGLU-PRO-LYS-DSER-THR-DLYS-DPRO-ASN-DGLU
Design116427	ASP-GLU-SER-DLEU-TYR-DALA-PRO-DPRO-TRP-ALA-ASN-DASP-DPRO-DARG-LEU
Design120532	LYS-ASN-VAL-DGLN-DSER-ASP-LYS-DARG-DVAL-DPRO-DPRO-DLEU-DPRO-DASP-DTYR
Design16897	THR-DSER-LYS-DLYS-PRO-DASP-DGLU-DARG-DALA-DASN-ASN-DSER-GLU-PRO-DSER
Design32090	GLU-ALA-PRO-DARG-DPRO-DLEU-GLU-DPRO-TYR-DGLN-DTYR-DSER-DASN-ASN-DSER
Design17434	DLEU-DARG-GLU-DTYR-DLEU-DASP-DGLU-PRO-PRO-GLU-LYS-ALA-DGLN-DASN-DASP
Design185692	GLU-PRO-LYS-DTHR-DSER-DGLN-DALA-DARG-DLYS-DPRO-PRO-ASP-ASP-ARG-DALA
Design136805	PRO-PRO-GLU-DSER-DALA-GLN-DSER-DSER-DGLU-DSER-PRO-DPRO-ALA-DLYS-DVAL
Design166081	ALA-THR-ASN-DSER-DGLN-DLYS-DPRO-PRO-DASP-DALA-DTHR-DILE-THR-DLYS-ASP
Design167736	PRO-DPRO-SER-DGLU-DPRO-DSER-DSER-DPRO-GLU-DALA-LYS-DALA-DPRO-ASN-DSER
Design2599	GLU-GLU-DASN-GLU-DALA-SER-DTYR-DGLU-PRO-PRO-DTHR-DLYS-DGLU-DVAL-ASP

Top 20 Residue Designs	
**Name**	**Sequence**
Design24037	DLYS-DSER-DSER-THR-ASN-DGLU-DASN-GLU-DALA-ARG-DGLU-DPRO-PRO-DILE-LEU-DGLU-DSER-DGLU-ASN-DALA
Design80837	PRO-DPRO-DPRO-ALA-ALA-ASN-GLU-SER-DSER-THR-DASP-DGLU-DTHR-DALA-ASN-DASN-ASN-DASN-LYS-DGLU
Design25226	DLYS-HIS-DLYS-PRO-SER-DLYS-ASP-LEU-LYS-DGLU-DALA-DGLN-PRO-TYR-DSER-DSER-DASN-ASP-ALA-LYS
Design119115	DASN-PRO-DGLN-DALA-LYS-DALA-ASN-DTHR-TYR-ASP-ASP-ARG-DARG-SER-DGLU-DALA-GLU-DSER-GLN-DPRO
Design22588	THR-DGLU-PRO-DPRO-DALA-GLU-ASP-GLU-DALA-DARG-GLU-SER-LEU-DALA-DLYS-PRO-DVAL-DHIS-DLYS-DLEU

Design74102	DALA-DLYS-DHIS-DPRO-ASN-GLU-DASN-THR-SER-GLU-ALA-DGLN-DGLU-DPRO-DARG-ASN-DGLU-PRO-ALA-DASP
Design143166	DASN-ASP-LEU-SER-DALA-DARG-DPRO-PRO-DGLU-GLN-DPRO-DTHR-LYS-ASP-GLU-DTHR-GLN-LYS-DSER-SER
Design45902	DLYS-HIS-DLYS-PRO-THR-DLYS-ASP-PHE-LYS-DGLU-DALA-DGLN-PRO-TYR-DSER-DSER-DASN-ASP-ALA-LYS
Design26034	DLYS-HIS-DLYS-PRO-THR-DGLU-LYS-PHE-LYS-DGLU-DALA-DGLN-PRO-TYR-DSER-DSER-DASN-ASP-ALA-LYS
Design1665	DALA-ASP-ASP-ALA-PRO-DASP-DALA-DSER-ALA-ASP-LYS-DTHR-DMET-DPRO-DPRO-LYS-DASP-DGLU-DARG-THR
Design111	TYR-DHIS-DGLU-DHIS-DASP-DGLU-DSER-THR-DTYR-LYS-DTHR-DGLU-PRO-DTHR-DPRO-ALA-DGLN-GLU-DTHR-DASN
Design83218	ASP-ARG-DASN-DLYS-PRO-DLYS-DASP-DPRO-DVAL-TYR-DGLU-PRO-DPRO-DASN-DALA-DASN-DLYS-DSER-TYR-ASN
Design107505	DLYS-DPRO-ASN-DTYR-ASN-DPRO-DLYS-DLEU-SER-DGLU-PRO-DASN-DTHR-DASN-GLU-DPRO-GLU-DARG-DTHR-DALA
Design59754	ALA-DTHR-DTHR-DGLU-DTHR-DSER-DSER-DTYR-PRO-PRO-ASP-GLU-DLYS-THR-DTHR-DSER-DASN-GLU-DVAL-DHIS
Design12800	DPRO-PRO-DARG-DPHE-DASN-ASN-DGLN-ILE-PRO-DASN-SER-DLYS-DPRO-DASP-DLEU-DGLN-ASP-LEU-SER-DGLU
Design15036	TYR-PRO-DGLU-DALA-DSER-SER-LYS-DASP-DASP-DALA-DGLU-ASP-PRO-DLYS-DALA-DARG-DLYS-DLEU-GLN-DSER
Design27893	DGLN-SER-DPRO-SER-ASP-GLN-SER-PRO-LYS-DLYS-ASN-DASP-DGLU-PRO-LEU-DSER-DASP-DGLU-DTYR-DASN
Design68384	SER-GLU-DASN-ALA-LYS-DASP-DLEU-DGLU-PRO-DILE-DALA-DPRO-DASN-TYR-DPRO-DTHR-DPRO-LYS-DALA-PRO
Design23193	SER-GLU-ALA-DLYS-DASN-DALA-DPRO-PRO-DSER-DPRO-DSER-DASP-DPRO-DSER-LYS-DASN-DGLU-PRO-VAL-DTYR
Design35869	PRO-ASP-ALA-ASN-DGLU-ASP-MET-DALA-ALA-DSER-ILE-LYS-DGLU-GLU-DGLU-ASN-DSER-DARG-DPRO-DGLU
Design101360	ASP-LYS-DSER-DLEU-LYS-DLYS-DASP-DASP-DVAL-DASP-DALA-DASP-DGLU-PRO-DVAL-ALA-LYS-PRO-ASN-DGLU
Design17670	THR-SER-PRO-DALA-LYS-DASP-DLEU-GLU-DLYS-DASN-THR-DLYS-DASP-DALA-DPRO-DPRO-DARG-TYR-DALA-DGLU

Top 24 Residue Designs	
**Name**	**Sequence**
Design759	LYS-DASP-DASP-DLEU-DLYS-ASN-DLEU-DTHR-DGLU-DPRO-LEU-DASN-PRO-DILE-TYR-SER-ASN-DGLN-DALA-ALA-DLYS-DASN-DALA-GLU
Design10052	SER-DGLU-ALA-DPRO-DTHR-DALA-DALA-ALA-DPRO-PRO-DILE-GLU-LEU-DSER-DTHR-ASP-ALA-THR-ASN-DALA-ASN-DASP-DLYS-DASN
Design32190	PRO-ASP-LYS-DASP-DALA-PRO-DASN-DTHR-DGLU-DPRO-DHIS-DGLU-DTYR-DGLU-DPRO-DLYS-PRO-DASN-ALA-DTYR-DGLU-DGLU-ALA-DLYS
Design21660	THR-ALA-DALA-ALA-DALA-PRO-DGLU-DASN-DHIS-LYS-DPRO-DSER-DGLN-DPRO-DALA-GLU-DASN-GLU-DGLU-DTHR-THR-DASP-DLEU-DLYS
Design36199	ALA-SER-DVAL-DSER-DGLU-THR-DVAL-DGLU-DPRO-DASP-DLYS-DPRO-GLU-DLYS-DALA-DTHR-PRO-DALA-DALA-ASN-DTHR-DASP-DALA-DSER
Design16647	ASN-DGLN-SER-SER-DTHR-TYR-DALA-THR-DASP-DPRO-DTHR-PRO-DLEU-DALA-DTHR-DGLU-PRO-DASP-DARG-DARG-ALA-DGLN-ASP-ALA
Design37605	DASP-DLYS-DARG-DALA-DARG-DASN-DALA-DGLU-DTHR-DGLU-SER-DALA-ASP-LEU-PRO-DASN-DSER-GLU-LYS-DASP-DLYS-PRO-THR-ALA
Design31759	SER-DTHR-SER-SER-DGLU-LYS-DTYR-THR-DASP-DTYR-DARG-PRO-DLYS-DALA-DPRO-DPRO-PRO-DASP-DGLN-DALA-ALA-DGLN-ASP-MET
Design25212	SER-GLU-THR-DGLU-SER-DHIS-PRO-DSER-SER-ARG-DASN-ASP-ASP-GLN-DASN-LEU-GLN-DALA-LEU-DVAL-PRO-DALA-THR-DASN
Design15225	ALA-ASN-DTHR-ALA-DTHR-DSER-THR-DLYS-TYR-DASN-ASN-DASP-DALA-DGLU-DVAL-DTHR-PRO-DSER-GLU-PRO-LYS-DGLU-DASN-ASN
Design2901	DSER-LYS-DGLU-PRO-DTYR-DASN-DVAL-DGLU-ASN-SER-DGLU-DGLN-DSER-DLEU-ARG-ASP-GLU-DGLU-LYS-DALA-DVAL-PRO-DASN-SER
Design18496	THR-ASN-DLEU-DALA-DGLN-ALA-ARG-DTHR-DTHR-DASP-DILE-DTYR-DLYS-DPRO-PRO-THR-DARG-DPRO-SER-DALA-ASP-GLN-GLU-DLYS
Design20199	DPRO-ARG-DGLU-DGLN-DTHR-TYR-DGLU-DASP-DVAL-THR-THR-SER-DGLU-DPRO-GLU-DLYS-PRO-DLYS-DALA-DALA-THR-DSER-DLEU-DLYS
Design27953	THR-ASP-HIS-DSER-DGLU-PRO-SER-DLYS-SER-DALA-DASP-DGLU-DARG-DLYS-DTYR-PHE-ASP-DHIS-DARG-PRO-DGLN-DGLU-LYS-DPRO
Design19384	DTYR-LEU-PRO-GLU-LEU-DSER-ALA-GLN-DGLU-PRO-ALA-DTHR-ALA-DASP-DLYS-DARG-DALA-DGLU-DPRO-DARG-SER-DSER-THR-DTYR
Design21698	DSER-THR-PRO-ALA-LYS-SER-ASN-DVAL-PRO-LEU-DASP-DARG-DALA-DLYS-HIS-ASP-ASP-LYS-DARG-ASP-PRO-DGLN-DALA-ASN
