# Supplementary material for: Heuristic energy-based cyclic peptide design
Source: PLoS Comput Biol. 2025 Apr 30;21(4):e1012290. doi: 10.1371/journal.pcbi.1012290 (PMC12043242; doi:10.1371/journal.pcbi.1012290)
Supplement: S9 Text — (PDF) [file pcbi.1012290.s009.pdf]

Response to Reviewers for *"Heuristic energy-based cyclic peptide design"*

February 3, 2025

Dear Dr. Slusky and Dr. Ben-Tal,

Thank you for handling our paper yourselves and for your helpful comments. Enclosed is our revision addressing the substantive comments along with point by point responses. Your comments regarding Figure Files and Data Requirements have also been taken care of.

In brief, we have conducted two additional validations to verify the structural stability of our designed cyclic peptides: (1) structural predictions for existing PDB cyclic peptides using our ClusterGen energy landscape sampling algorithm; (2) replica exchange MD simulations starting from alternative backbone conformations for the designed amino acid sequences.

Accurate structural predictions and consistent free energy surfaces give evidence that the proposed top designs are promising candidates for future experiments.

Moreover, we have made four smaller changes: (1) corrected a typo in two amino acid abbreviations found in the MD/REMD simulations; (2) showed an efficiency comparison between CyclicChamp and Rosetta for 20- and 24-residue designs; (3) updated our Introduction to reflect the current state of the literature on cyclic peptide design; (4) listed amino acid sequences of our designs.

We hope you will find the revision acceptable for publication in *PLOS Computational Biology*.

Warm Regards,

Dennis Shasha

Response to Reviewer 1

The paper titled "Heuristic Energy-Based Cyclic Peptide Design" introduces a new computational pipeline, CyclicChamp, designed to efficiently generate de novo cyclic peptides. This innovative approach marks a significant improvement over previous random sampling methods by employing simulated annealing, making it faster and more capable of handling larger macrocycles of up to 24 amino acids. The research combines molecular dynamics simulations to validate the kinetic and thermodynamic stability of the peptides, leading to promising candidates for future experimental evaluation.

Strengths are:
1. Innovative Methodology
2. Relevant Applications
3. Accurate computational validation
4. Efficiency and scalability

Reply: Thank you! You have understood our goals completely.

However, the manuscript completely lacks any experimental validation. Therefore, I believe the manuscript should be accepted only after - some - experimetal validation is provided.

Reply: We agree. While chemical synthesis and experimental evaluation of a new peptide would be beyond the scope of the present work, we have now added computational validations of previously-published experimentally-determined peptide macrocycle structures found in the Protein Data Bank. Using our new method, we are able to make blind predictions, based on sequences alone, of most previously-published macrocycles, without imposing symmetry constraints to bias simulations (as was previously necessary for peptides in the 12-24 residue range using the old Rosetta methods). Please see our response to the editorial board below for more details.

Response to Editorial board

> Though the method is novel, which should be sufficient for publication here without experiments, the results should be benchmarked compared to known structures. There should be quite a few cyclic peptides of known structure, and for this manuscript to be validated, we would want to see the RMSD of the model vs real for enough of these (perhaps 20?). It seems like Table 1 touches on this, but it is not clear enough nor does it have enough examples.

Reply: Thank you for this constructive suggestion. We have now provided structural predictions for 20 existing PDB macrocycle structures that have relevant lengths of 7-24 residues and no cross-links. The results are shown in Figure 9 in the main text, and Figure S18 and S19 in the supplementary. The methodology is added in the subsection "Structure predictions for PDB macrocycles" in Materials and Methods, and text describing the analysis is added in the subsection "Structure predictions for existing PDB macrocyles" in Results.

The predictions are produced using our stability analysis algorithm, ClusterGen, which samples the energy landscape and identifies low-energy structures. With only the amino acid sequence as the input, accurate predictions of < 1.5 Å RMSD from the experimental structures are achieved for 15 out of the 20 cases. In the remaining five cases, two predictions are still good considering the relatively large macrocycle sizes (1.836 Å RMSD for a 12-mer and 1.559 Å for a 24-mer). Notably, the large peptides' structures were predicted in an unbiased fashion (*i.e.* without imposing any prior knowledge, such as symmetry expectations). This is in contrast with past work (such as Mulliagn *et al.* (2020) *Protein Sci.* 29(12):2433-45), in which structure prediction was carried out with a biased algorithm sampling only symmetric conformations in order to make the prediction problem tractable. The other three inaccurate predictions (2-3 Å) result from the inherently unstable PDB structures, having either two distinct crystal forms, or long, floppy loop regions shown in NMR models.

Hence, our ClusterGen algorithm is capable of thoroughly sampling the rugged energy landscapes and serves as an effective validation tool for structural stability analysis. The designs that pass the P_{Near} stability analysis are good candidates for future experiments. We hope that this blind prediction of past experimental results sufficiently addresses this point.

> I understand that you are already using MD for validation. I think for MD to be a validation tool, I'd like to see simulations with wrong cyclic peptide structures. And I'd like to see that the simulations are different enough from the right structure to be able to tell right from wrong (For 20 cases).

Reply: This is a good point. In addition to the previous 6 REMD simulations for randomly permutated sequences as negative controls (Table 1 and Figure S16), we now include 12 REMD simulations starting from "wrong" backbone conformations. By performing the same REMD simulation protocol, we can see whether the free energy surfaces (FES) generated still converge to the original ones or not. If so, this would validate that our REMD has done sufficient sampling and is not biased by the starting point. The results are shown in Figure S16 (b) and S17 (b), and text analysis is added to the subsection "Replica Exchange results for stable trajectories" in Results.

We want to highlight that the 15-residue design 169032 stands out as its new FES is almost identical to the original, further confirming its strong stability and our strong confidence in the MD validation. Among the other four top designs (Table 1), the 15-residue design 136805 and the 20-residue 68384 also show a similar new FES compared with the original. Hence, we label these three designs *computationally validated.*

Because the new FES of the experimentally solved structures 6ucx, 6uf7, and 6uf8 did not converge well with the original ones, instead of suggesting that these are unstable, we think such failure of FES to converge from

different starting points represents an ambiguous result: here, the computational validation is unable to give an definitive conclusion. Hence, we label the 15-residue Design 17434 and the 24-residue 20199 *computationally suggestive*. These labels (*computationally validated*, *computationally suggestive*, and *failed* design) are listed in Table 1 for readers' convenience.

> Finally, though I understand AF/ESM/RoseTTAFold don't yet handle D-amino acids, if any of your designs are all L, could you please validate the structure using your structure prediction method of choice?

Reply: Thank you for the suggestion. Unfortunately, all of our designs are heterochiral (*i.e.* they contain both L- and D-amino acids), which prevents application of the current ML-based methods. The D-amino acid counts are shown in Figure S7 in the supplementary information. We have, however, added some text to the introduction describing the state of the art in ML-based peptide modelling, and the need for methods compatible with heterochiral peptides (such as our CyclicChamp) which is unmet by ML models. We have also added text to the eighth paragraph of the Results section mentioning that all of our designs are heterochiral. We also added the following text to the start of the "Molecular dynamics simulations for top designs" section, explaining why MD was used in lieu of ML methods: "We next sought a means of validating designs that was independent of Rosetta or the conformational sampling methods developed here. Since currently-available ML methods for predicting macrocycle structures are incompatible with heterochiral peptides, we turned to molecular dynamics (MD) simulations."

Notes on other changes made:

1. During our revision, we found there was an typographical error in two of the D-amino acid abbreviations in the inputs to some of our validation simulations: when sending designed structures for MD and REMD simulations, we needed to change from four letter abbreviations to three letters, and for two amino acids, D-Asparagine and D-Glutamine, their abbreviations went from DASN and DGLN to DAS and DGL by our script, which caused them to be incorrectly recognized as D-Aspartate and D-Glutamate rsepectively.

For all designs impacted by this typo, we re-ran their MD and REMD simulations. While most simulation results remained similar, we were able to find one more stable design for 15 residues, as well as one for 24 residues. All relevant results and text are corrected now: Figure 8 and Table 1 in the main, Figure S10-S17 in the supplementary.

2. Before in our computation efficiency comparison, we ran only Rosetta's *simple_cycpep_predict* for designing 7- and 15-residue macrocycles. For completeness, we have now run Rosetta for 20- and 24-residue designs as well. The full comparison is available in Figure 2 now. For these large sizes, our CyclicChamp demonstrates much higher efficiency than Rosetta, taking only one-eighth or one-third of the computation time to find a stable design on average.

3. In the past months, several ML-based methods for designing and predicting cyclic peptides have been published in journals or posted in preprint. We include and cite them in our Introduction to keep updated.

4. Previously, amino acids identities were labeled on the structures in the figures, but the labels and order were a bit hard to discern. Now, the amino acid sequences for our good designs are listed in Table S2 in the supplementary. In the main text Figure 3-8, the designed sequences are also printed alongside structures shown there. We hope this is a bit clearer.

5. We have also made minor edits here and there. All changes are marked in red in the revised manuscript.

Thank you again for granting us additional time to revise our manuscript, enabling us to run more computations and validations.
